# Time-series transcriptomic analysis of cigarette smoke–associated lung responses reveals COPD-related inflammatory and epithelial remodeling modules in murine models

**DOI:** 10.3389/fmed.2026.1785075

**Published:** 2026-06-24

**Authors:** Meng Long, Anhuizi Qiu, Wangyu Jiang, Fangya Guo, Siyi Tu, Fang Chen

**Affiliations:** 1 The First Clinical Medical College of Zhejiang Chinese Medical University, Hangzhou, China; 2 Respiratory Physiology Laboratory, The First Affiliated Hospital of Zhejiang Chinese Medical University, Hangzhou, China

**Keywords:** bioinformatics, candidate genes, chronic obstructive pulmonary disease, cigarette smoke, time-series transcriptomics

## Abstract

**Background:**

Cigarette smoke is the major environmental risk factor for chronic obstructive pulmonary disease (COPD), driving progressive lung inflammation and tissue injury. However, distinguishing general smoke-responsive transcriptional changes from COPD-related molecular signatures remains challenging.

**Methods:**

Public time-series transcriptomic datasets of cigarette smokeokemains challenging. and tissue injury. ity, Han-related chronic airway inflammation model (GSE132661) were analyzed. Principal component analysis (PCA), differential expression analysis, gene set enrichment analysis (GSEA), protein–protein interaction (PPI) network analysis, and functional enrichment were performed to characterize stage-dependent transcriptional dynamics. Candidate genes were further evaluated using receiver operating characteristic (ROC) analysis, quantitative real-time PCR (qPCR), and exploratory analysis in a human COPD transcriptomic dataset.

**Results:**

PCA revealed progressively increased transcriptomic divergence with prolonged smoke exposure. GSEA demonstrated a shift from early epithelial differentiation and barrier-related alterations to activation of pro-inflammatory and immune migration pathways. Six consistently upregulated genes were identified. Among them, CD177 and KRT85 showed stable expression changes across time points and models, with qPCR confirming significant upregulation after 2 and 5 months of smoke exposure (*P* < 0.001). Exploratory human dataset analysis suggested heterogeneous and severity-dependent expression patterns.

**Conclusion:**

This integrative time-series analysis identifies CD177 and KRT85 as murine model-derived candidate genes associated with cigarette smokeeverity confirming significant upregulation after 2 and 5 tial expression analysis, gene ance in human COPD remains exploratory, heterogeneous, and severity-dependent, and further validation in larger, clinically well-characterized cohorts is required.

## 1 Introduction

Chronic obstructive pulmonary disease (COPD) is a common respiratory disorder with a substantial global disease burden. According to the Global Burden of Disease study, the number of individuals living with COPD exceeded 400 million worldwide in 2019, resulting in approximately 3.23 million deaths and ranking COPD as the third leading cause of death globally (1). Beyond its high prevalence and mortality, COPD imposes a considerable socioeconomic and healthcare burden, with the impact being particularly pronounced in low- and middle-income countries (2). Cigarette smoking is widely recognized as the most important risk factor and primary etiological cause of COPD (3). Epidemiological evidence indicates that approximately 45% of COPD cases in men and 25% in women can be attributed to long-term cigarette smoke exposure (1). Consequently, smoking-induced chronic pulmonary inflammation and tissue injury are regarded as central mechanisms underlying the initiation and progression of COPD.

It is well established that cigarette smoke contains thousands of toxic chemical compounds as well as high concentrations of reactive oxygen species (ROS), which impose persistent oxidative stress and inflammatory stimuli on the respiratory tract. Moreover, long-term smoking disrupts the oxidant-antioxidant balance within the lung, thereby triggering oxidative stress-mediated molecular damage and chronic inflammatory responses (4). Accumulating evidence has demonstrated significantly elevated levels of oxidative damage markers—such as lipid peroxidation products, 8-isoprostanes, and nitrotyrosine—in the airways, lung tissues, and peripheral blood of patients with COPD (5). These oxidative stress-related injuries and the ensuing inflammatory responses are mediated through multiple signaling pathways, among which nuclear factor-κB (NF-κB) plays a pivotal role. Cigarette smoke exposure activates the NF-κB signaling pathway and promotes its nuclear translocation, leading to the transcriptional upregulation of a wide array of downstream pro-inflammatory genes, including cytokines and chemokines, thereby driving chronic airway inflammation (6). This process results in excessive production of neutrophil chemoattractants (such as IL-8/CXCL8) and pro-inflammatory cytokines (including TNF-α and IL-6) (6).

In addition to activating inflammatory signaling cascades, cigarette smoking also stimulates both innate and adaptive immune responses. Specifically, smoking induces bronchial epithelial cells to secrete granulocyte-macrophage colony-stimulating factor, promotes the accumulation of macrophages and neutrophils, and activates immune cell subsets such as Th17 cells and CD8∧ + T lymphocytes (7). Increasing attention has been directed toward the role of interleukin-17 (IL-17), secreted by Th17 cells, in COPD airway inflammation. Elevated IL-17 levels have been observed in the lungs of patients with severe COPD and are closely associated with neutrophilic infiltration and lymphoid follicle formation (8).Collectively, cigarette smoke exerts a dual pathogenic effect through oxidative stress and inflammatory pathways, leading to persistent airway mucosal inflammation and progressive tissue destruction, thereby laying the foundation for COPD development. Notably, smoking-induced airway inflammation may persist even after smoking cessation (5). Despite cessation, alterations in both innate and adaptive immune responses remain active in the lungs, manifesting as sustained inflammatory cell infiltration and aberrant cytokine expression. This phenomenon of “inflammatory memory” suggests that smoking-related chronic injury can become self-perpetuating and further accelerate COPD progression. These observations underscore the need for an in-depth investigation of the temporal effects of cigarette smoke exposure on lung tissue, in order to elucidate the mechanistic links between early disease-triggering events and the chronic, often irreversible, inflammatory processes observed at later stages.

In recent years, high-throughput transcriptomic technologies have been increasingly applied to elucidate the molecular mechanisms underlying COPD. In population-based studies, gene expression profiling of airway epithelial cells and peripheral blood from patients with COPD has revealed that cigarette smoking induces widespread transcriptomic alterations, including the upregulation of genes related to inflammatory mediators, proteases, and mucus hypersecretion. However, given that COPD develops through a long-term cumulative process, cross-sectional studies at a single time point are insufficient to capture the dynamic nature of disease progression. To address this limitation, time-series transcriptomic studies using animal models have provided a unique and valuable perspective. For example, Miller et al. conducted a longitudinal RNA sequencing study in mice exposed to cigarette smoke for up to 9 months and demonstrated that even a single day of smoke exposure was sufficient to trigger acute antioxidant and detoxification responses. In contrast, prolonged exposure over several months led to dysregulation of distinct biological pathways, including pyrimidine metabolism, phosphatidylinositol signaling, and lysosomal function, ultimately resulting in marked impairments in pulmonary energy metabolism, phagocytic capacity, and DNA repair pathways (9). Notably, they also identified several previously unreported smoking-associated differentially expressed genes, such as Lox (lysyl oxidase, involved in extracellular matrix remodeling) and Gp2 (a goblet cell-associated protein), indicating that chronic smoke exposure affects matrix reconstruction and mucus secretion (9). Moreover, a subset of genes upregulated in smoke-exposed mice exhibited similar expression trends in lung tissues from patients with COPD, including elastase MMP12, osteoinductive protein GPNMB, soluble differentiation antigen CD68, and the chemokine CCL22 (10). Collectively, these findings suggest that transcriptomic alterations observed in animal models can, to a considerable extent, recapitulate the pathological features of COPD in humans.

Nevertheless, a systematic comparison of transcriptomic profiles across distinct exposure time points remains lacking, particularly with respect to delineating the molecular trajectory underlying the transition from acute injury to chronic inflammation. Based on this critical knowledge gap, the present study investigated gene expression changes in lung tissues following short-term (1 day), intermediate-term (2 months), and long-term (5 months) cigarette smoke exposure in a mouse model, and explored their associations with COPD-related chronic airway inflammation through integrative bioinformatic analyses. Gene expression microarrays were used to profile lung transcriptomes at each time point, followed by the identification of differentially expressed genes (DEGs), construction of protein-protein interaction (PPI) networks, and functional enrichment analyses to identify key pathways and central regulatory genes. In addition, selected candidate genes that emerged prominently from the analyses (e.g., Cd177 and Krt85) were subjected to quantitative real-time PCR (qPCR) validation and receiver operating characteristic (ROC) curve analysis to explore their association with smoke-induced inflammatory phenotypes. Compared with previous studies, the major innovation of this work lies in its systematic characterization of the dynamic evolution of cigarette smoke-induced pulmonary transcriptomic changes over time, thereby bridging molecular events from acute responses to chronic inflammatory stages. This integrative temporal framework contributes to a deeper understanding of smoke-induced chronic airway inflammatory remodeling and provides a transcriptomic basis for future mechanistic investigation.

## 2 Materials and methods

### 2.1 GEO datasets

This study primarily utilized two publicly available datasets from the Gene Expression Omnibus (GEO) database to systematically investigate transcriptomic alterations in lung tissues during time-dependent cigarette smoke exposure and to explore their biological relevance to COPD-related chronic airway inflammation. All datasets were downloaded from the GEO database.^1^ We selected the mouse lung gene expression microarray dataset GSE18344 (platform: GPL1261, Affymetrix Mouse Genome 430 2.0 Array), which profiles lung tissues from wild-type (WT) and Nrf2 knockout (KO) mice under different cigarette smoke exposure conditions (11). The experimental design of GSE18344 includes multiple variables, including genotype (WT vs. Nrf2 knockout), treatment (sham vs. smoke), smoke dose (low, medium, and high), exposure duration (1 day, 2 months, and 5 months), and recovery conditions.

To minimize potential confounding effects arising from genotype heterogeneity and dose variation, the primary time-series analysis in the present study was restricted to WT mice exposed to a consistent medium-dose cigarette smoke condition and their matched sham controls. Specifically, the following subsets from GSE18344 were included: WT-sham-1 day (GSM457913-GSM457916), WT-smoke-med-1 day (GSM457917-GSM457920), WT-sham-2 month (GSM457921-GSM457924), WT-smoke-med-2 month (GSM457925-GSM457928), WT-sham-5 month (GSM457937-GSM457940), and WT-smoke-med-5 month (GSM457945-GSM457948). Samples from Nrf2 knockout mice, low- or high-dose smoke exposure groups, and recovery groups were excluded from the primary analyses. Thus, the final primary time-series dataset consisted of 24 WT samples, including 12 sham controls and 12 smoke-exposed samples across three representative time points (1 day, 2 months, and 5 months). A complete sample annotation and selection table for GSE18344 is provided in Supplementary Table S1.

To validate and assess the robustness of smoke-associated transcriptional responses related to chronic airway inflammation, an independent GEO dataset representing a COPD-related chronic airway inflammation model, GSE132661 (platform: GPL7202, Agilent-014868 Whole Mouse Genome Microarray 4 × 44K), was included as an external validation cohort. In this dataset, chronic pulmonary inflammation was induced in mice through repeated intratracheal instillation of lipopolysaccharide (LPS), resulting in a persistent inflammatory airway microenvironment. Only samples from the LPS-treated group and the phosphate-buffered saline (PBS) control group were included in the present analysis to construct transcriptional features associated with COPD-related chronic airway inflammation.

**FIGURE 1:**
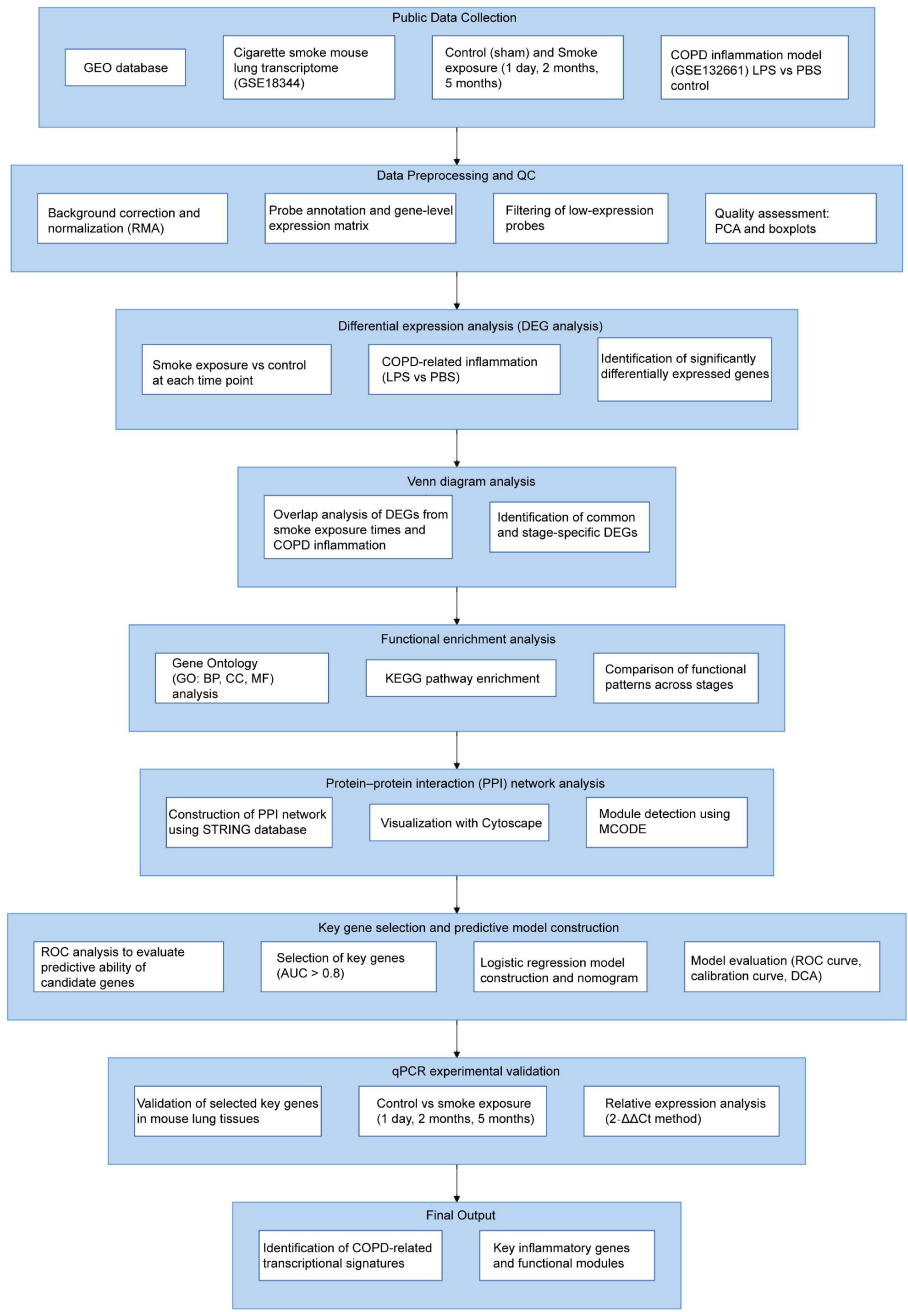
Schematic overview of the analytical workflow used in this study.

Raw expression data (CEL files or normalized expression matrices) for both datasets were downloaded from the GEO database using the GEOquery package in R. Following data acquisition, uniform preprocessing and quality control procedures were applied separately to the cigarette smoke exposure time-series dataset and the COPD-related chronic airway inflammation validation dataset. The inclusion of GSE132661 as an external validation dataset was intended to verify candidate genes and functional modules identified during cigarette smoke exposure in an independent chronic inflammation model, thereby enabling discrimination between general smoke-responsive genes and transcriptional responses that are stably maintained under chronic airway inflammatory conditions and potentially closely associated with COPD initiation and progression (12). An overview of the subsequent data analysis workflow is presented in Figure 1.

### 2.2 Data preprocessing and quality control

All data preprocessing and quality control procedures were performed using R software. For the Affymetrix microarray platform (GPL1261), raw CEL files were preferentially downloaded and processed whenever available. If only normalized expression matrices were provided, consistency checks were conducted and the processing workflow was harmonized as much as possible across datasets. Background correction, normalization, and probe summarization were performed using the affy or oligo packages, and the Robust Multi-array Average (RMA) algorithm was applied to generate log2-transformed expression matrices, thereby reducing technical noise and improving cross-sample comparability.

For GSE18344, preprocessing was conducted after sample selection for the primary analysis, which included only the 24 WT samples under sham or medium-dose smoke exposure at 1 day, 2 months, and 5 months. This restriction was applied before downstream analysis to ensure comparability across time points and to reduce confounding introduced by genotype- and dose-related heterogeneity.

To minimize background interference caused by low-intensity probes, low-expression probes were filtered out (note that the FPKM concept is not applicable to microarray platforms). Specifically, the filtering strategy involved: (1) calculating the distribution of expression intensities and variability for each probe across all samples; (2) removing probes that consistently exhibited low expression levels in the majority of samples and showed minimal variability, based on expression quantile thresholds or filtering approaches implemented in the genefilter package; and (3) annotating probes to gene symbols using the GPL1261 annotation file. When multiple probes mapped to the same gene, either the average expression value was calculated or the probe with the highest variance was selected as the representative, yielding a gene-level expression matrix for downstream analyses.

For quality control, principal component analysis (PCA) was performed to assess the overall variance structure of the dataset and clustering patterns among samples, enabling the identification of potential outliers. In addition, boxplots of normalized expression values were generated to evaluate normalization performance and ensure consistency of expression distributions across samples. If batch effects were detected (e.g., arising from different experimental or scanning batches), they were corrected using the ComBat method implemented in the sva package, or alternatively controlled by including batch variables as covariates in the design matrix for differential expression analysis. PCA and other quality control assessments for GSE18344 were performed on the selected WT medium-dose time-series subset to confirm sample comparability and detect potential outliers before differential expression and enrichment analyses.

### 2.3 Differentially expressed gene (DEG) analysis

Differential expression analyses were performed to identify genes associated with distinct stages of cigarette smoke exposure as well as COPD-related chronic airway inflammation. For the cigarette smoke exposure time-series dataset (GSE18344), differential expression analyses were restricted to the selected WT mice exposed to a consistent medium-dose cigarette smoke condition and their matched sham controls at each time point (1 day, 2 months, and 5 months), as described in section 2.1. Specifically, the following comparisons were conducted: (1) cigarette smoke exposure groupsom-day exposure vs. control, 2-month exposure vs. control, and 5-month exposure vs. control; and (2) the COPD-related chronic airway inflammation modelons -induced chronic airway inflammation group vs. phosphate-buffered saline (PBS) control group.

Differential expression analysis was carried out using the limma package in R. Design matrices were constructed according to the experimental design, incorporating treatment conditions and exposure time points. For the cigarette smoke exposure dataset, contrasts were defined between each exposure time point and the corresponding control group to identify stage-specific DEGs. For the COPD-related chronic airway inflammation dataset, contrasts were defined between the LPS-treated group and the control group to extract DEGs associated with the chronic inflammatory phenotype. Linear models were fitted to the expression data using the lmFit function, followed by empirical Bayes moderation of the standard errors using the eBayes function, thereby improving the stability and reliability of differential expression estimates under limited sample sizes. Significantly differentially expressed genes were identified based on adjusted *P*-values (false discovery rate, FDR < 0.05), corresponding to the adj.P.Val output generated by the limma package using the Benjamini–Hochberg multiple testing correction method, together with |log2 fold change| ≥ 1. DEG lists were generated for each comparison based on these criteria. To visualize global expression patterns under different experimental conditions, DEG results were further subjected to graphical analyses. Volcano plots were generated to display the distribution of significantly upregulated and downregulated genes for each comparison. All statistical significance shown in the volcano plots was based on adjusted *P*-values (FDR). In addition, the top 50 most significant DEGs ranked by adjusted *P*-value from each comparison were selected to construct heatmaps, illustrating their expression patterns and hierarchical clustering across samples, thereby providing an intuitive overview of transcriptomic alterations associated with time-dependent cigarette smoke exposure and chronic airway inflammation.

Gene set enrichment analysis (GSEA) was performed using a pre-ranked strategy in R (version 4.2.1). For each comparison, genes were ranked according to the moderated t-statistics generated by the limma package. Pre-ranked GSEA was conducted using the clusterProfiler package with 10,000 permutations and Benjamini–Hochberg correction for multiple testing. The canonical pathway collection from MSigDB (c2.cp.all.v2022.1.Hs.symbols.gmt), which integrates pathway annotations from Reactome, KEGG, and WikiPathways, was used as the reference gene set database. Mouse gene symbols were mapped to pathway gene sets based on symbol matching. The minimum and maximum gene set sizes were set to 10 and 500, respectively. For each enriched pathway, the normalized enrichment score (NES), nominal *P* value, adjusted q value (FDR), rank position, leading-edge subset, and core enrichment genes were extracted for downstream interpretation. Enrichment results were visualized using the ggplot2 package (version 3.4.4). The complete GSEA results for all comparisons are provided in Supplementary Table S3, and the full lists of differentially expressed genes (DEGs) for each comparison are available in Supplementary Table S4. To account for potential cell-type composition bias in bulk transcriptomic data, marker-based proxy scores were calculated for major immune cell populations (neutrophils, macrophages, and T cells) using curated gene sets. These scores were incorporated into sensitivity analyses and included as covariates in adjusted differential expression models.

To further account for potential cell-type composition bias in bulk lung transcriptomic data, a marker-based proxy score analysis was performed for major immune cell populations, including neutrophils, macrophages, and T cells. For each cell type, curated marker gene sets were defined based on established literature, and proxy scores were calculated as the mean normalized expression (Z-score) of the corresponding marker genes across samples. These proxy scores were subsequently incorporated into sensitivity analyses and included as covariates in adjusted differential expression models to evaluate the robustness of the observed transcriptomic patterns.

### 2.4 Venn diagram analysis of differentially expressed genes

Venn diagram analysis was performed using the VennDiagram package to compare overlaps among differentially expressed genes (DEGs) identified at distinct stages of cigarette smoke exposure (1 day, 2 months, and 5 months) and those identified in the COPD-related chronic airway inflammation model. This approach was used to identify gene subsets that dynamically respond to cigarette smoke exposure while exhibiting consistent expression changes under chronic inflammatory conditions, as well as stage-specific DEGs unique to individual exposure time points.

Specifically, DEG lists were first extracted separately for the 1-day, 2-month, and 5-month cigarette smoke exposure groups, as well as for the COPD-related chronic airway inflammation group (LPS-induced group vs. control). These four DEG lists were then treated as independent gene sets and input into the VennDiagram tool to generate four-set Venn diagrams, enabling intuitive visualization of shared and unique DEG distributions across exposure stages and the COPD inflammation model.

Based on the Venn diagram results, particular attention was focused on genes that were consistently upregulated or downregulated across multiple cigarette smoke exposure time points and concurrently altered in the COPD-related chronic airway inflammation model. These shared DEGs were considered potential core transcriptional features associated with COPD and were subsequently subjected to downstream functional enrichment analyses and mechanistic investigations. In addition, UpSet analysis was performed to quantitatively characterize intersection patterns among DEG sets.

### 2.5 Protein-protein interaction (PPI) network construction and module analysis

#### 2.5.1 Construction of the protein-protein interaction (PPI) network

Protein-protein interaction (PPI) information was obtained from the Search Tool for the Retrieval of Interacting Genes/Proteins (STRING) database. Based on the candidate differentially expressed gene sets identified through differential expression and Venn diagram analyses, the gene set used for PPI construction consisted of differentially expressed genes that overlapped between cigarette smoke exposure at each time point (1 day, 2 months, and 5 months) and the COPD-related chronic airway inflammation model. Specifically, DEGs from each exposure stage were intersected with the COPD model DEGs, and the union of these COPD-related intersecting gene sets was used as the input for PPI analysis, rather than being restricted to the small subset of genes shared across all exposure stages. Based on the candidate differentially expressed gene sets identified through differential expression and Venn diagram analyses—nalysesll subset of genes shared across all exposure stages. Based exposure and the COPD-related chronic airway inflammation modelall exposure stages. Based exposure and the to the STRING database to retrieve PPI information. A confidence score threshold was applied (e.g., combined score ≥ 0.7) to retain high-confidence protein-protein interactions. Interaction data, including protein pairs and their corresponding interaction scores, were downloaded from STRING and imported into Cytoscape software for visualization. Only interactions among the input genes were retained for network construction, without introducing additional external interactors, to ensure consistency between the PPI network and the defined intersecting DEG set. This approach enabled the construction of PPI networks and intuitive visualization of interaction relationships among candidate genes, as well as characterization of network topology features.

#### 2.5.2 PPI module identification and functional enrichment analysis

Based on the constructed PPI networks, functional modules were identified using the MCODE plugin in Cytoscape, which detects highly interconnected subnetworks with dense connectivity. These modules were considered to represent core gene clusters that may act cooperatively during COPD-related chronic airway inflammation. For each module identified by MCODE, functional enrichment analyses were subsequently performed to explore their potential biological significance. Gene symbols within each module were converted to Entrez IDs, and Gene Ontology (GO) enrichment analyses—nalyses) enrichment Onn biologiP), cellular component (CC), and molecular function (MF) categories—as well as Kyoto Encyclopedia of Genes and Genomes (KEGG) pathway enrichment analyses were conducted using the clusterProfiler package in R. Adjusted *P* < 0.05 were applied as the significance threshold to identify enriched biological processes and signaling pathways. Enrichment results for each module were visualized using bar plots or bubble plots, facilitating interpretation of key biological functions and pathways associated with the identified gene clusters and providing a foundation for subsequent mechanistic analyses.

### 2.6 Candidate gene selection and exploratory discriminative analysis

To evaluate the exploratory discriminative ability of candidate genes for identifying COPD-related chronic airway inflammation, receiver operating characteristic (ROC) curve analysis was systematically applied to genes within the PPI network modules. The pROC package in R was used to calculate the area under the ROC curve (AUC) for each gene in discriminating between the COPD-related chronic airway inflammation group and the corresponding control group. Genes with an AUC value greater than 0.8 were defined as candidate key genes with good discriminative performance and were selected for subsequent predictive model construction.

Based on the selected key genes, logistic regression models were constructed to assess their predictive value for the risk of COPD-related chronic airway inflammation. Using the regression coefficients derived from the logistic models, nomograms were further developed to translate gene expression levels into quantitative risk scores, enabling individualized risk prediction. To comprehensively evaluate the performance and reliability of the predictive models, ROC curves were generated to assess overall discriminative ability, while calibration curves were plotted to examine the agreement between predicted probabilities and observed outcomes. In addition, decision curve analysis (DCA) was performed to evaluate the net clinical benefit of the models across a range of risk thresholds, thereby providing an integrated assessment of the potential applicability and stability of the key gene-based predictive models for COPD-related chronic airway inflammation risk evaluation.

### 2.7 Quantitative real-time PCR (qPCR) experimental design

(1) Candidate gene selection: Based on the results of differential expression analysis, time-series expression pattern analysis, PPI network construction, and ROC curve analysis, 5–10 candidate genes with clear biological relevance and statistical significance were comprehensively selected for subsequent qPCR validation. The selection criteria were as follows: (i) genes closely associated with chronic airway inflammation, immune cell recruitment, or epithelial stress responses; (ii) genes exhibiting stable and directionally consistent expression changes across different stages of cigarette smoke exposure and in the COPD-related chronic airway inflammation model; and (iii) genes demonstrating significance and concordance across multiple analytical strategies, including DEG analysis, time-series analysis, PPI network analysis, and ROC-based evaluation.

(2) Experimental materials: qPCR validation was performed using mouse lung tissue samples. These samples were obtained from an independent mouse experiment conducted at the Animal Experimental Center of Zhejiang Chinese Medical University between August 2022 and June 2024. Experimental groups included a control group and cigarette smoke–exposed groups at 1 day, 2 months, and 5 months. All animals were wild-type (WT) mice, and each group consisted of *n* = 10 biological replicates. Cigarette smoke exposure was performed using a custom-built whole-body exposure chamber (0.85 m × 0.7 m × 0.7 m). Commercial cigarettes (Hongtashan; tar 10 mg, nicotine 0.9 mg, carbon monoxide 11 mg) were used as the smoke source. The concentration of smoke particles was maintained at approximately 300.0 ± 25.0 mg/mł, including both mainstream and sidestream smoke. Each exposure session consisted of 20 cigarettes and lasted for 1 h. Mice were exposed twice daily with a 4-h interval between sessions. The exposure protocol was conducted continuously for 16 weeks. On the day of LPS administration, cigarette smoke exposure was withheld to avoid acute interaction effects. This protocol was adapted based on previously reported cigarette smoke exposure models and optimized under laboratory conditions. Samples for qPCR validation were collected at 1 day, 2 months, and 5 months following the initiation of cigarette smoke exposure. Cigarette smoke exposure was performed under controlled laboratory conditions, while control animals were maintained under identical conditions without smoke exposure. Total RNA was extracted using RNAiso Plus (TaKaRa, Japan), and reverse transcription was performed using the PrimeScript™ RT reagent Kit with gDNA Eraser (TaKaRa, Japan). Quantitative PCR was conducted using TB Green^®^ Premix Ex Taq™ II (Tli RNaseH Plus, TaKaRa, Japan) according to the manufacturer’s instructions. Gene-specific primers were designed and validated using publicly available databases or Primer-BLAST to ensure specificity and amplification efficiency. Primer sequences are provided in Supplementary Table S2. Glyceraldehyde-3-phosphate dehydrogenase (GAPDH) was used as the internal reference gene to normalize variations in RNA input among samples. The stability of GAPDH expression across all experimental groups was systematically evaluated prior to analysis and showed no significant variation, supporting its suitability as a reference gene under the present experimental conditions. Although the use of multiple reference genes is generally recommended for improved normalization accuracy, the stability assessment performed in this study indicates that GAPDH provided reliable normalization for the current experimental design.

(3) Experimental procedures and data analysis: Following collection, mouse lung tissues were immediately snap-frozen in liquid nitrogen and stored at −80 °C until further processing. Fresh tissues were rinsed with physiological saline to remove residual blood and cut into small pieces (approximately 3–5 mm in size, thickness < 0.5 cm) prior to cryopreservation. Total RNA was extracted using RNAiso Plus according to the manufacturer’s instructions, and RNA concentration and purity were assessed using a NanoDrop ND-2000 spectrophotometer (Thermo Fisher Scientific, United States). Equal amounts of RNA were reverse-transcribed into complementary DNA (cDNA). Quantitative PCR was performed in a total reaction volume of 20 μL, containing 10 μL TB Green Premix Ex Taq II, 0.8 μL forward primer (10 μM), 0.8 μL reverse primer (10 μM), 2 μL cDNA template, and 6.4 μL RNase-free water. Amplification was carried out on a real-time PCR system (e.g., Stratagene, United States) under the following conditions: initial denaturation at 95°C for 30 s, followed by 40 cycles of 95°C for 5 s and 60°C for 30 s, with a subsequent melting curve analysis. Relative gene expression levels were calculated using the 2^∧^−ΔΔCt method, with GAPDH serving as the internal reference gene. Each sample was analyzed with at least three technical replicates. Data are presented as mean ± standard deviation (SD) from *n* = 10 biological replicates per group. Statistical analyses were performed using GraphPad Prism or R software. Differences among multiple groups were evaluated using one-way analysis of variance (ANOVA), followed by Tukey’s *post hoc* multiple comparison test. A *P* < 0.05 was considered statistically significant. Finally, qPCR results were compared with bioinformatic findings to assess the consistency and reliability of candidate gene expression changes, thereby further validating their potential roles in cigarette smoke exposure-induced lung injury and COPD-related chronic airway inflammation.

### 2.8 Exploratory validation in a human COPD transcriptomic dataset

To further explore the translational relevance of the candidate genes in human COPD, we analyzed the GSE47460 dataset using samples from the GPL14550 platform. Samples annotated as interstitial lung disease were excluded. COPD and control samples were defined according to the clinical annotations provided in GEO. Because GSE47460 includes patients with heterogeneous COPD severity, an exploratory severity-stratified analysis was additionally performed by comparing GOLD 4 very severe COPD samples with control samples. The processed sample-level dataset used for this exploratory GOLD 4 COPD versus control analysis is provided in Supplementary Table S5. For transparency, the corresponding processed sample-level dataset for the all-COPD versus control comparison in GSE47460-GPL14550 is provided in Supplementary Table S6. CD177 and KRT85 expression values were extracted according to platform-specific probe annotations. The probe annotation and mapping information for CD177 and KRT85 in the GPL14550 platform are provided in Supplementary Table S7. For the GOLD 4 versus control comparison, differential expression was evaluated using linear models with adjustment for age, sex, and smoking status. Z-score transformation was used only for visualization and not for statistical testing. The primary interpretation was based on model-derived coefficients and *P*-values. This analysis was considered exploratory and was not intended to establish CD177 and KRT85 as definitive diagnostic biomarkers for human COPD.

### 2.9 Ethics statement

All animal experiments in this study were conducted in strict accordance with the Regulations for the Administration of Laboratory Animals of the Peoplen this study were conducted in strict accotocol was reviewed and approved by the Ethics Committee of the institution (Approval No.IACUC-20220228-07). All procedures were performed under the supervision of trained personnel to ensure animal welfare and full compliance with ethical guidelines.

## 3 Results

### 3.1 Data preprocessing and quality control

**FIGURE 2:**
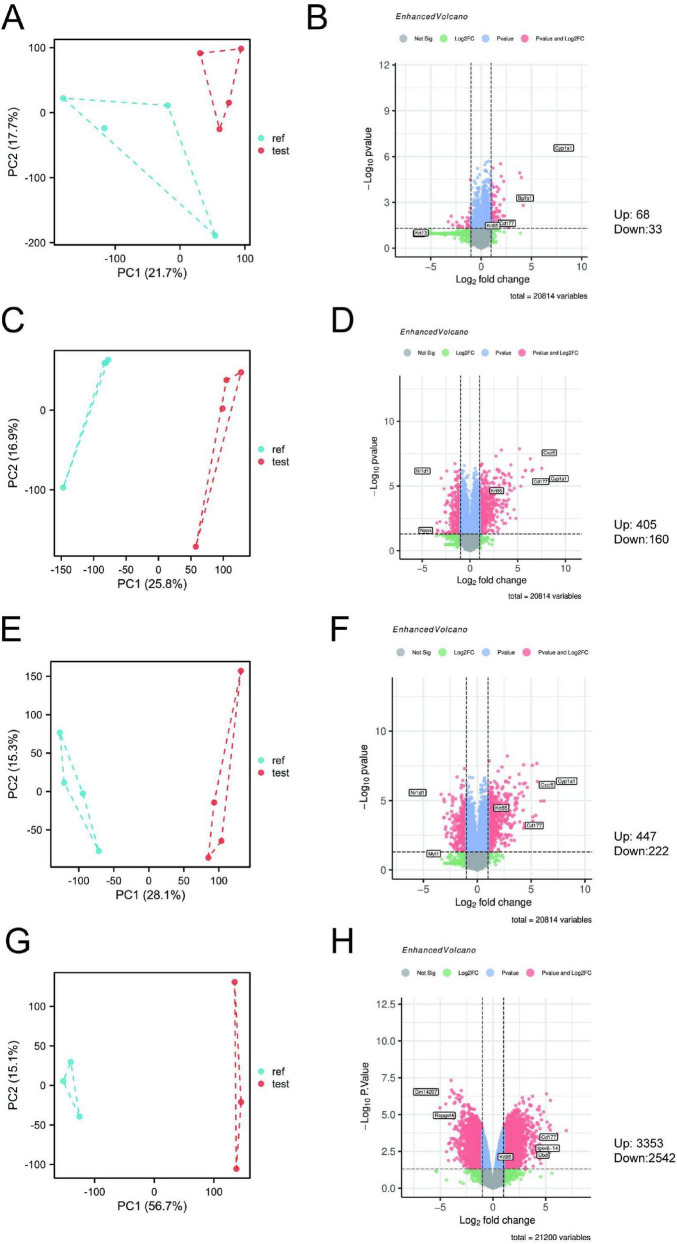
Differential expression analysis of gene expression data across cigarette smoke exposure time points and LPS-induced chronic airway inflammation models. **(A)** PCA analysis for 1-day exposure. **(B)** Volcano plot for 1-day exposure with selected top differentially expressed genes labeled. **(C)** PCA analysis for 2-month exposure. **(D)** Volcano plot for 2-month exposure with selected genes labeled. **(E)** PCA analysis for 5-month exposure. **(F)** Volcano plot for 5-month exposure with selected genes labeled. **(G)** PCA analysis for LPS-induced chronic airway inflammation group. **(H)** Volcano plot for the LPS group with selected genes labeled.

As shown in Figure 2, principal component analysis (PCA) and differential expression visualization were used to evaluate data quality and global transcriptomic variation across different exposure conditions. In the PCA analysis of the 1-day cigarette smoke exposure group (Figure 2A), samples from the smoke-exposed group (test) and the control group (ref) exhibited clear separation in principal component space, indicating that short-term smoke exposure was sufficient to induce detectable transcriptomic alterations at the global level. Consistently, the corresponding volcano plot (Figure 2B) revealed a subset of significantly upregulated and downregulated genes at this early time point, suggesting rapid activation of early stress- and inflammation-related transcriptional responses. The boxplots of normalized gene expression intensities, demonstrating consistent expression distributions across samples, are provided in Supplementary Figure S1A.

In the 2-month cigarette smoke exposure group, PCA results (Figure 2C) showed a more pronounced separation between smoke-exposed and control samples along both PC1 and PC2 axes, suggesting that transcriptomic differences progressively accumulated and intensified with prolonged exposure. The corresponding volcano plot (Figure 2D) demonstrated a marked increase in the number of differentially expressed genes, with clearly separated distributions of upregulated and downregulated genes along the fold-change axis, reflecting extensive transcriptional remodeling induced by chronic smoke exposure. The corresponding normalized expression boxplots are shown in Supplementary Figure S1B, confirming stable expression distributions across samples.

In the 5-month cigarette smoke exposure group, PCA analysis (Figure 2E) revealed an almost complete separation between smoke-exposed and control samples in principal component space, indicating that long-term continuous exposure established robust and stable transcriptomic differences at the global expression level. The corresponding volcano plot (Figure 2F) displayed a large number of significantly upregulated and downregulated genes, suggesting activation of transcriptional programs associated with sustained inflammation, tissue remodeling, and functional dysregulation. Normalized expression boxplots for this group are presented in Supplementary Figure S1C.

Finally, in the LPS-induced chronic airway inflammation model (COPD-like inflammation validation group), PCA analysis (Figure 2G) also demonstrated clear separation between the experimental (test) and control (ref) groups, indicating that a chronic inflammatory microenvironment alone can induce substantial global transcriptomic alterations. The corresponding volcano plot (Figure 2H) revealed a considerable number of differentially expressed genes; however, the overall distribution pattern differed to some extent from that observed in the long-term cigarette smoke exposure group, suggesting that inflammation-driven transcriptional responses may exhibit distinct characteristics. The normalized signal intensity boxplots for the LPS model are provided in Supplementary Figure S1D.

### 3.2 Stage-dependent expansion of differentially expressed genes with exposure duration

**FIGURE 3:**
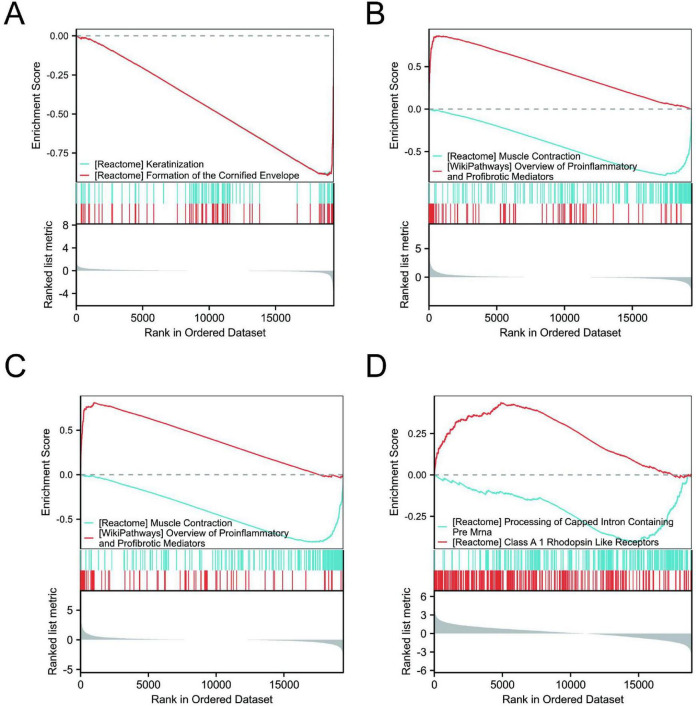
Pre-ranked gene set enrichment analysis (GSEA) illustrating significantly enriched pathways across different stages of cigarette smoke exposure and in a COPD-related chronic airway inflammation model. Genes were ranked by limma moderated t-statistics, and enrichment was re-evaluated using 10,000 permutations. Representative pathways are shown with their normalized enrichment scores (NES), nominal *P*-values, and adjusted q values. **(A)** Early-stage cigarette smoke exposure—GSEA analysis. **(B)** Mid-stage cigarette smoke exposure—GSEA analysis. **(C)** Late-stage cigarette smoke exposure—GSEA analysis. **(D)** LPS-induced chronic airway inflammation group—GSEA analysis.

Gene set enrichment analysis (GSEA) of differentially expressed genes (DEGs) is summarized in Figure 3. Pre-ranked GSEA was performed using limma moderated t-statistics as the ranking metric with 10,000 permutations, and the enrichment patterns remained stable compared with the initial analysis, supporting the robustness of the results. In the early stage of cigarette smoke exposure, GSEA results (see Figure 3A) showed that significantly enriched pathways were predominantly related to keratinization, including Keratinization and Formation of the Cornified Envelope. Specifically, REACTOME_KERATINIZATION showed significant negative enrichment (NES = −2.42, nominal *P* = 1.0 × 10^−10^, *q* = 1.16 × 10^−7^), and REACTOME_FORMATION_OF_THE_CORNIFIED_ENVELOPE showed similar negative enrichment (NES = −2.36, nominal *P* = 1.0 × 10^−10^, *q* = 1.16 × 10^−7^). The leading-edge subsets included representative epithelial structural genes such as Ppl, Klk12, Klk5, Dsp, Perp, Tgm1, Krt14, Krt5, Lor, and Krt4. These pathways were significantly enriched on the negative side of the ranked gene list, indicating that gene programs associated with epithelial differentiation and barrier structure were markedly suppressed during the initial phase of smoke exposure. This finding suggests that airway epithelial homeostasis is disrupted at a very early stage following smoke-induced stress. The complete GSEA results are provided in Supplementary Table S3.

In the intermediate stage of cigarette smoke exposure, GSEA results (see Figure 3B) exhibited a more pronounced inflammation-associated signature. The Overview of Proinflammatory and Profibrotic Mediators pathway was significantly enriched on the positive side of the ranked gene list, whereas the Muscle Contraction pathway was enriched on the negative side. These enrichment patterns remained consistent after re-analysis using 10,000 permutations, confirming that the observed pathway shifts were not driven by stochastic variation in gene ranking. These patterns indicate that with prolonged smoke exposure, pro-inflammatory and pro-fibrotic molecular networks become progressively activated, while genes involved in normal tissue contraction and structural maintenance are concurrently downregulated.

In the late stage of cigarette smoke exposure, these enrichment trends were further amplified (Figure 3C). Pathways related to pro-inflammatory and pro-fibrotic mediators continued to show strong positive enrichment, whereas negative enrichment of the Muscle Contraction pathway became more pronounced. Re-analysis with 10,000 permutations yielded consistent enrichment direction and statistical significance, further supporting the reproducibility of late-stage transcriptional remodeling. This pattern suggests that long-term smoke exposure is associated with sustained amplification of inflammatory signaling, accompanied by persistent suppression of transcriptional programs related to tissue function and structural integrity. Collectively, these results reflect a transcriptomic shift under chronic smoke exposure from early inflammatory activation toward gene expression patterns linked to functional impairment and tissue remodeling.

In the LPS-induced chronic airway inflammation model, GSEA results (see Figure 3D) revealed enrichment of pathways including Class A/1 Rhodopsin-like Receptors and Processing of Capped Intron-Containing Pre-mRNA. Receptor-related signaling pathways were significantly enriched on the positive side of the ranked gene list, whereas pathways associated with RNA processing were enriched on the negative side. These enrichment signals were also preserved after permutation-based re-analysis, indicating that the identified pathways are robust features of the chronic inflammatory transcriptional landscape. These findings indicate that within a chronic inflammatory microenvironment, pathways involved in cellular signal transduction and inflammatory sensing are activated, while components of basal post-transcriptional processing undergo coordinated remodeling. This enrichment pattern is consistent with transcriptional regulatory alterations characteristic of sustained inflammatory states.

### 3.3 Cell-type composition analysis and robustness evaluation

To assess whether the observed transcriptomic changes were influenced by shifts in cell-type composition in bulk lung tissue, we performed a marker-based proxy score analysis for major immune cell populations, including neutrophils, macrophages, and T cells. As shown in Supplementary Figures S2A–C, neutrophil proxy scores were significantly increased at the intermediate stage (2 months), while macrophage proxy scores were elevated at both intermediate and late stages (2 and 5 months), indicating progressive immune cell infiltration during chronic smoke exposure. In contrast, T cell proxy scores remained relatively stable across all time points. To further address potential confounding effects from non-immune cellular components, we extended the proxy score analysis to include epithelial and fibroblast/stromal cell populations. Epithelial proxy scores (based on EPCAM, CDH1, and keratin family genes) showed significant differences between smoke-exposed and control groups at early (1 day) and late (5 months) stages, whereas fibroblast/stromal proxy scores (based on COL1A1, COL1A2, COL3A1, DCN, and related markers) did not exhibit significant changes across exposure time points (Supplementary Figures S2D,E).

To further validate these findings, we applied the same analysis to the LPS-induced chronic airway inflammation dataset. Consistent increases in macrophage and T cell proxy scores were observed in the LPS group compared with controls (Supplementary Figures S2D–F), supporting the relevance of immune cell dynamics in chronic inflammatory conditions. In contrast, epithelial and fibroblast/stromal proxy scores did not show statistically significant differences between LPS-treated and control samples (Supplementary Figures S2G–J). Importantly, after incorporating proxy scores for both immune (neutrophils, macrophages, and T cells) and non-immune (epithelial and fibroblast/stromal) cell populations as covariates in the differential expression models, the global transcriptomic patterns remained broadly comparable. Strong correlations were observed between original and proxy-adjusted gene-level statistics across all time points (Supplementary Figures S2K–M), indicating that the main findings were not solely driven by changes in immune, epithelial, or stromal cell composition. Nevertheless, the stage-dependent variation in epithelial proxy scores observed in the cigarette smoke exposure model suggests that epithelial cell composition or epithelial cell-state changes may contribute to part of the differential gene expression signals detected in bulk lung tissue, particularly for genes and pathways related to epithelial differentiation, keratinization, and barrier remodeling. Therefore, the smoke-induced DEG profiles should be interpreted as reflecting both cell-intrinsic transcriptional regulation and changes in epithelial cellular composition or state during disease progression.

### 3.4 Venn diagram and UpSet analysis of differentially expressed genes

**FIGURE 4:**
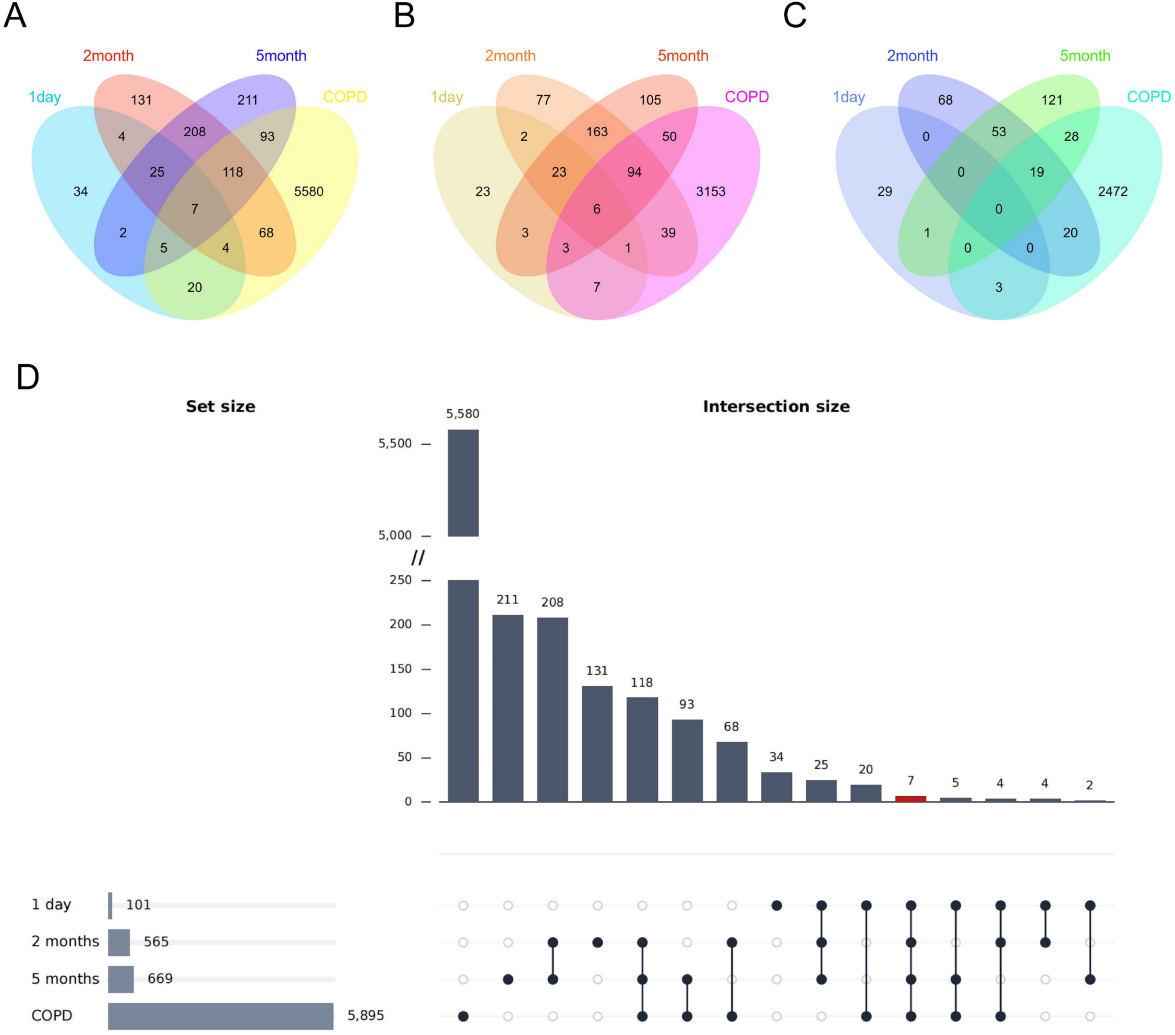
Venn diagram analysis illustrating overlaps of total **(A)**, upregulated **(B)** and downregulated **(C)** differentially expressed genes (DEGs) among cigarette smoke exposure at different time points and a COPD-related chronic airway inflammation model. **(A)** Overlap of all DEGs among smoke exposure at 1 day, 2 months, 5 months, and COPD-related chronic airway inflammation. **(B)** Overlap of commonly upregulated DEGs across smoke exposure time points and COPD-related chronic airway inflammation. **(C)** Overlap analysis of downregulated DEGs showing no commonly downregulated genes across conditions. **(D)** UpSet plot showing the size and distribution of DEG intersections across all conditions, including both unique and shared gene sets among different exposure stages and the COPD-related chronic airway inflammation model.

As shown in Figure 4, Figure 4A illustrates the overlap of all differentially expressed genes (DEGs) among different stages of cigarette smoke exposure (1 day, 2 months, and 5 months) and the COPD-related chronic airway inflammation model. Overall, both shared and stage-specific DEGs were observed among the smoke exposure groups and between the smoke exposure groups and the COPD inflammation model, indicating that cigarette smoke-induced transcriptional responses exhibit pronounced temporal dynamics. Notably, a subset of genes displayed stable expression changes that were maintained across multiple exposure stages and persisted under chronic inflammatory conditions.

Further Venn diagram analysis focusing on upregulated genes (Figure 4B) identified six genes that were consistently upregulated across all cigarette smoke exposure stages and in the COPD-related chronic airway inflammation model: CYP1B1, CD177, GCH1, BTBD11, KRT85, and ADAM8. These genes exhibited sustained upregulation during early, intermediate, and late phases of smoke exposure and showed concordant upregulation in the COPD inflammation model, suggesting that they may represent a stable transcriptional signature associated with persistent inflammatory states. Functionally, these genes are mainly involved in oxidative stress responses and xenobiotic metabolism (CYP1B1, GCH1), neutrophil activation and inflammatory cell migration (CD177, ADAM8), epithelial stress responses and structural remodeling (KRT85), as well as transcriptional regulation and inflammation-related modulatory processes (BTBD11), collectively pointing to the coordinated activation of multilayered molecular networks within the chronic airway inflammatory microenvironment (Table 1). It should be noted that the set of commonly upregulated genes shared across all exposure stages represents a highly stringent subset of transcriptional features. While these genes are useful for identifying robust candidate biomarkers, such strict intersection criteria may exclude a substantial number of biologically relevant genes involved in coordinated inflammatory responses.

**TABLE 1:** Detailed information of core upregulated DEGs associated with cigarette smoke exposure and COPD-related chronic airway inflammation.

No.	Gene	Full name	Sign	Function
1	CYP1B1	Cytochrome P450 Family 1 Subfamily B Member 1	Up	A xenobiotic-metabolizing enzyme involved in oxidative stress responses and regulation of inflammatory signaling, frequently induced by cigarette smoke exposure.
2	CD177	CD177 Molecule	Up	A neutrophil surface glycoprotein associated with neutrophil activation, migration, and chronic airway inflammatory responses.
3	GCH1	GTP Cyclohydrolase 1	Up	The rate-limiting enzyme for tetrahydrobiopterin (BH4) biosynthesis, involved in nitric oxide production and inflammation-associated oxidative stress.
4	BTBD11	BTB Domain Containing 11	Up	A BTB/POZ domain-containing protein implicated in transcriptional regulation and cellular stress responses; its role may relate to inflammation-associated regulatory remodeling.
5	KRT85	Keratin 85	Up	A structural keratin protein reflecting epithelial cell stress, differentiation, or remodeling under chronic inflammatory conditions.
6	ADAM8	ADAM Metallopeptidase Domain 8	Up	A metalloprotease involved in leukocyte migration, extracellular matrix remodeling, and inflammation-driven tissue injury, frequently linked to chronic airway inflammation.

In contrast, Venn diagram analysis of downregulated genes (Figure 4C) did not identify any commonly downregulated DEGs shared across different cigarette smoke exposure stages and the COPD inflammation model. This observation suggests that transcriptional responses associated with chronic airway inflammation are primarily characterized by sustained upregulation of inflammation- and stress-related genes rather than by uniform gene repression, which is consistent with the molecular pathology of COPD as a disease driven by chronic and persistent inflammatory activation.

In addition, UpSet analysis was performed to quantitatively characterize the intersection patterns among DEG sets (Figure 4D and Supplementary Table S3). The results showed that the largest gene set was derived from the COPD-related chronic airway inflammation model (5,895 genes), which markedly exceeded those from the cigarette smoke exposure groups (1 day: 101 genes; 2 months: 565 genes; 5 months: 669 genes). Among the smoke exposure groups, the largest overlap was observed between the 2-month and 5-month groups (208 genes), followed by the overlap between the 5-month group and the COPD model (93 genes), and between the 2-month group and the COPD model (68 genes). In contrast, overlaps involving the 1-day exposure group were relatively small, indicating limited shared transcriptional changes at the early stage. The intersection shared across all four conditions was relatively small (7 genes), consistent with the stringent filtering observed in the Venn diagram. In addition, several intermediate-sized intersections were identified, including gene sets shared between three conditions, further illustrating the complexity of DEG distribution across exposure stages and the COPD model. Overall, the UpSet plot provides a quantitative overview of DEG overlap, highlighting both dominant and minor intersection patterns among the different conditions.

### 3.5 Protein-protein interaction network, functional enrichment analysis, and predictive performance evaluation

**FIGURE 5:**
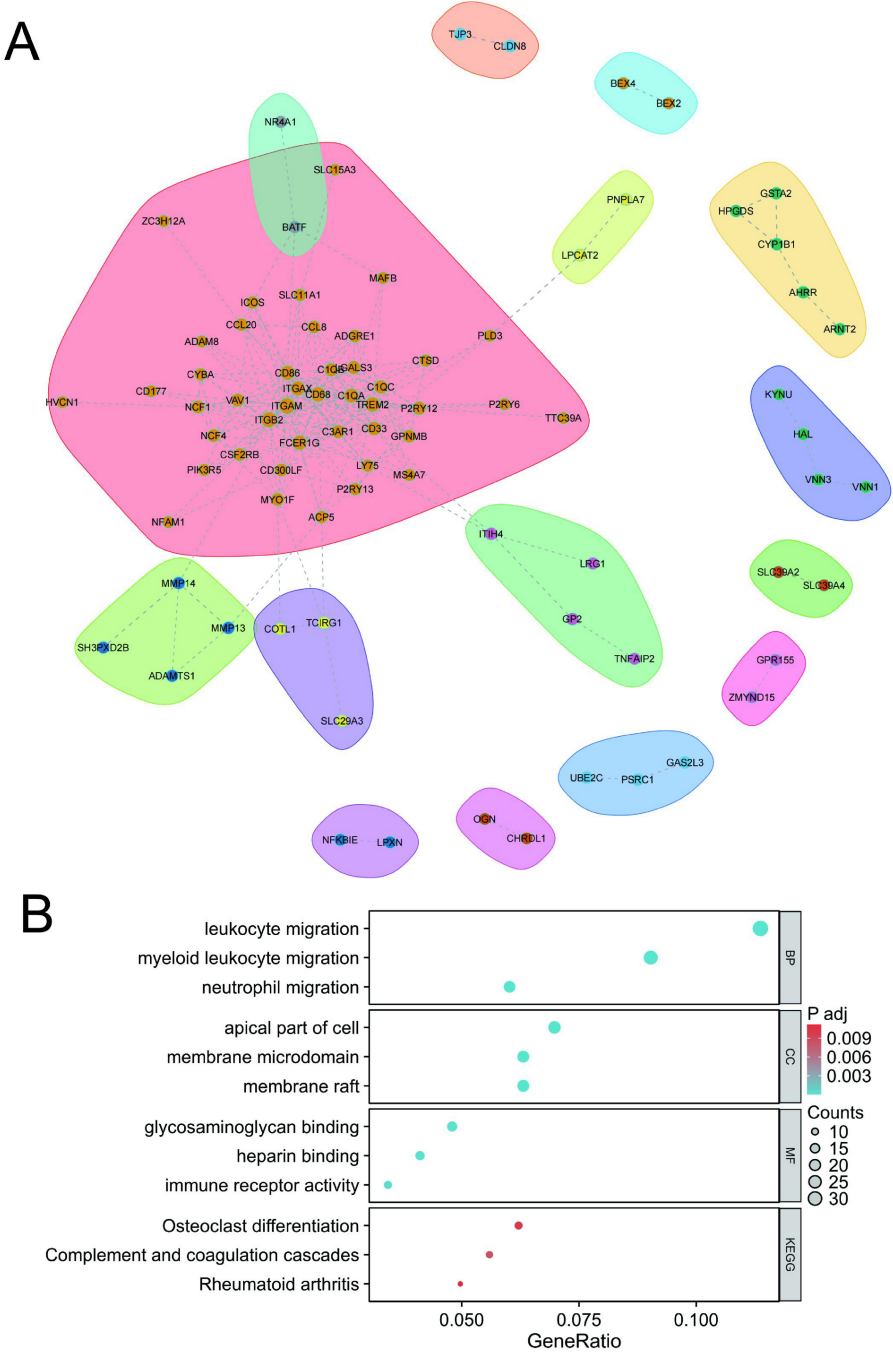
Protein-protein interaction (PPI) network and functional enrichment analysis of intersecting DEGs across cigarette smoke exposure stages and COPD-related chronic airway inflammation. **(A)** PPI network of intersecting DEGs constructed using the STRING database. **(B)** GO and KEGG enrichment analysis of all intersecting DEGs across smoke exposure time points and COPD.

The protein-protein interaction (PPI) network constructed using the STRING database is shown in Figure 5A. The network was constructed based on differentially expressed genes that overlapped between cigarette smoke exposure at each time point (1 day, 2 months, and 5 months) and the COPD-related chronic airway inflammation model, rather than only the small subset of genes shared across all exposure stages. Specifically, DEGs from each exposure stage were intersected with the COPD model DEGs, and the union of these COPD-related intersecting gene sets was used to construct the PPI network, allowing a more comprehensive representation of coordinated biological interactions. The results demonstrate that the intersecting differentially expressed genes shared among different stages of cigarette smoke exposure (1 day, 2 months, and 5 months) and the COPD-related chronic airway inflammation model form a densely connected interaction network. Within this network, multiple inflammation-related receptors, adhesion molecules, and signal transduction factors occupy central positions, suggesting that these genes may cooperatively participate in immune cell recruitment, amplification of inflammatory responses, and tissue reactions within the chronic airway inflammatory microenvironment.

Subsequent Gene Ontology (GO) and KEGG pathway enrichment analyses of all intersecting DEGs across the three exposure stages and the COPD model are summarized in Figure 5B. At the biological process (BP) level, these genes were significantly enriched in immune-related processes, including leukocyte migration, myeloid leukocyte migration, and neutrophil migration. In terms of cellular components (CC), enrichment was predominantly observed in membrane microdomains, membrane rafts, and the apical part of the cell. Molecular function (MF) analysis revealed significant enrichment in immune receptor activity, glycosaminoglycan binding, and heparin binding. KEGG pathway analysis further indicated close associations with immune and inflammatory pathways, such as complement and coagulation cascades, osteoclast differentiation, and rheumatoid arthritis, highlighting a strong functional concordance between smoke exposure-induced transcriptomic alterations and the molecular characteristics of COPD-related chronic inflammation.

**FIGURE 6:**
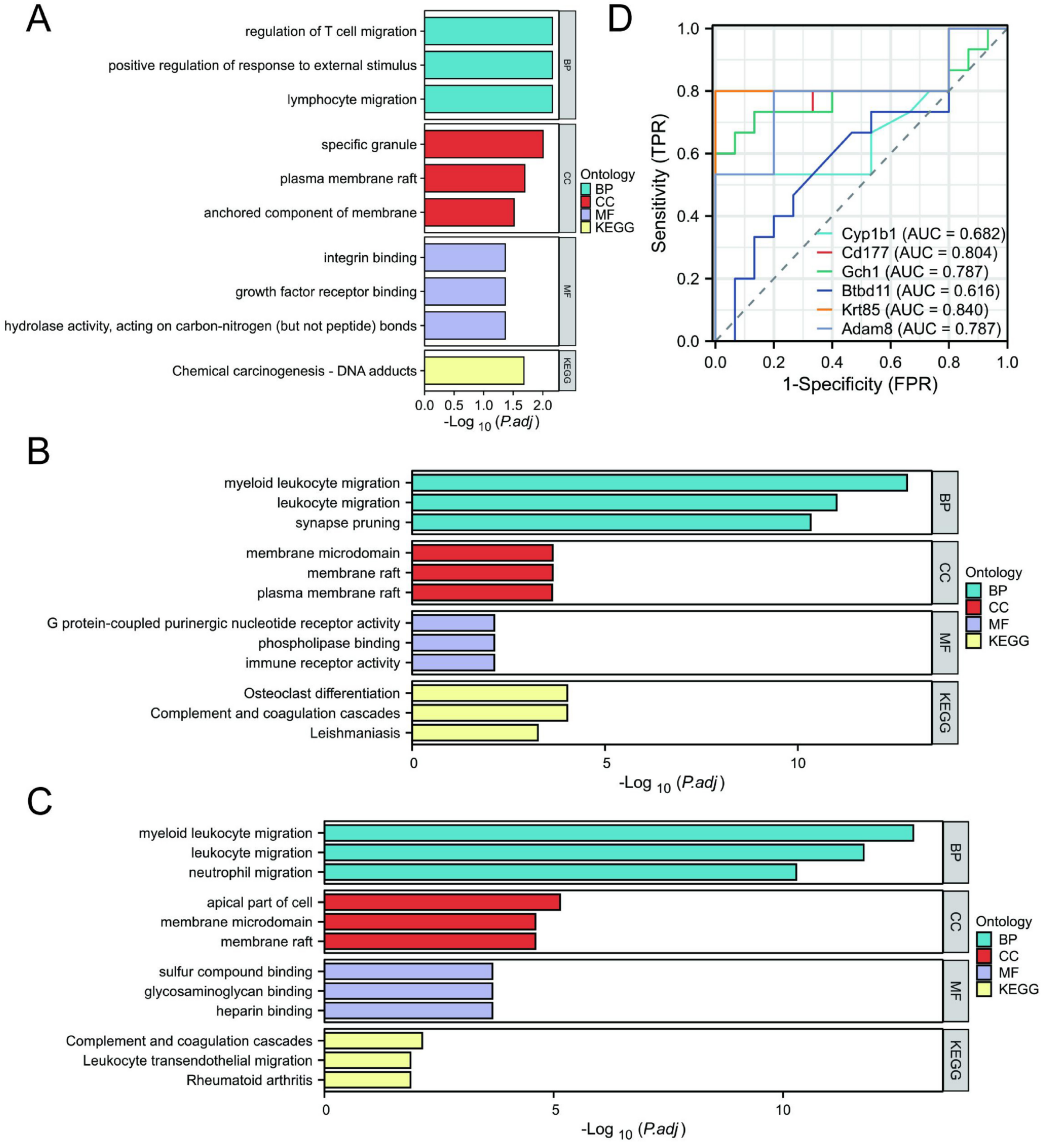
Stage-specific functional enrichment and ROC analysis of COPD-related intersecting DEGs. **(A)** GO and KEGG enrichment analysis of intersecting DEGs between 1 day smoke exposure and COPD. **(B)** GO and KEGG enrichment analysis of intersecting DEGs between 2 months smoke exposure and COPD. **(C)** GO and KEGG enrichment analysis of intersecting DEGs between 5 months smoke exposure and COPD. **(D)** ROC curve analysis evaluating the predictive performance of six commonly upregulated genes for LPS-induced chronic airway inflammation outcome.

To further delineate functional differences among intersecting genes at distinct exposure stages, stratified enrichment analyses were performed for genes shared between COPD and each exposure time point, with results shown in Figure 6. For genes common to the 1-day exposure group and the COPD model (Figure 6A), significantly enriched biological processes primarily involved regulation of T-cell migration, lymphocyte migration, and positive regulation of responses to external stimuli. These genes were enriched in plasma membrane rafts and membrane-anchored components at the cellular component level, while molecular functions included integrin binding and growth factor receptor binding. KEGG analysis revealed enrichment in DNA adduct formation pathways associated with chemical carcinogenesis, suggesting that early smoke exposure is sufficient to trigger molecular responses related to immune cell activation and environmental stimulus sensing.

In the analysis of genes shared between the 2-month exposure group and the COPD model (Figure 6B), biological process enrichment shifted toward myeloid leukocyte migration, leukocyte migration, and synapse pruning. Cellular component enrichment remained focused on membrane microdomains and membrane rafts, while molecular functions were enriched in G protein-coupled purinergic nucleotide receptor activity, phospholipase binding, and immune receptor activity. KEGG pathway analysis showed significant associations with complement and coagulation cascades, osteoclast differentiation, and infection-related pathways, indicating progressive expansion and amplification of inflammation-related signaling networks during intermediate-term smoke exposure.

For genes shared between the 5-month exposure group and the COPD model (Figure 6C), enrichment at the biological process level further emphasized core inflammatory processes, including myeloid leukocyte migration, leukocyte migration, and neutrophil migration. Cellular component enrichment consistently involved the apical part of the cell, membrane microdomains, and membrane rafts, while molecular functions were mainly associated with sulfur compound binding, glycosaminoglycan binding, and heparin binding. KEGG pathway analysis highlighted strong associations with leukocyte transendothelial migration, complement and coagulation cascades, and rheumatoid arthritis, suggesting that under long-term smoke exposure, transcriptomic features progressively converge toward a stable chronic inflammatory phenotype.

In addition, to evaluate the potential predictive value of the six consistently upregulated core genes in distinguishing COPD-related inflammatory states, ROC curve analyses were performed for CYP1B1, CD177, GCH1, BTBD11, KRT85, and ADAM8 (Figure 6D). These ROC analyses were performed based on the COPD-related chronic airway inflammation model (LPS vs. PBS), rather than clinical COPD samples. The results showed that several genes exhibited good discriminative performance between COPD and control conditions, including KRT85 (AUC = 0.840), CD177 (AUC = 0.804), GCH1 (AUC = 0.787), and ADAM8 (AUC = 0.787). CYP1B1 (AUC = 0.682) and BTBD11 (AUC = 0.616) also demonstrated moderate discriminative potential. Collectively, these findings suggest that this set of persistently upregulated inflammation-related genes may constitute a group of candidate genes associated with COPD-related chronic airway inflammatory responses in experimental models.

### 3.6 Exploratory discriminative model analysis based on CD177 and KRT85

**FIGURE 7:**
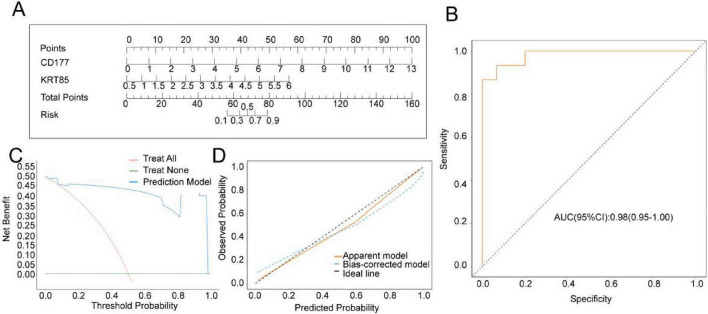
Exploratory model based on CD177 and KRT85 for distinguishing LPS-induced chronic airway inflammation from controls. **(A)** Nomogram constructed using the expression levels of CD177 and KRT85 for predicting the risk of COPD-related chronic airway inflammation. **(B)** Receiver operating characteristic (ROC) curve illustrating the sensitivity and specificity of the predictive model. **(C)** Decision curve analysis (DCA) demonstrating the net clinical benefit of the model across different risk thresholds. **(D)** Calibration curve assessing the agreement between predicted and observed probabilities of COPD-related chronic airway inflammation.

Based on the selected key genes CD177 and KRT85, a predictive model was further constructed to assess the risk of COPD-related chronic airway inflammation, and its predictive performance was systematically evaluated (Figure 7). The nomogram (Figure 7A) illustrates the relative contributions of CD177 and KRT85 expression levels within the model and their corresponding associations with risk of LPS-induced chronic airway inflammation. By assigning scores to the expression levels of the two genes and summing the total score, the individual probability of COPD occurrence can be directly estimated, highlighting the potential operability of this model for experimental or clinical stratification.

ROC curve analysis of the dual-gene model is shown in Figure 7B. The model demonstrated excellent discriminative performance in distinguishing LPS-induced chronic airway inflammation from controls, with an area under the curve (AUC) of 0.98 (95% CI: 0.95–1.00), indicating high sensitivity and specificity in reflecting disease states associated with chronic airway inflammation.

Decision curve analysis (DCA) was further applied to evaluate the net clinical benefit of the model (Figure 7C). Across a broad range of risk thresholds, the net benefit curve of the dual-gene model was consistently higher than those of the “treat-all” and “treat-none” strategies, suggesting potential applicability of the CD177-KRT85-based model across different risk levels. In addition, calibration curve analysis (Figure 7D) demonstrated good agreement between predicted probabilities and observed LPS-induced chronic airway inflammation outcomes, with the bias-corrected calibration curve closely approximating the ideal reference line. This finding indicates satisfactory calibration performance and model stability. Collectively, these results demonstrate that the dual-gene predictive model based on CD177 and KRT85 exhibits strong discriminative ability and reliability for assessing the risk of COPD-related chronic airway inflammation.

### 3.7 qPCR validation supports the expression consistency of key candidate genes

**FIGURE 8:**
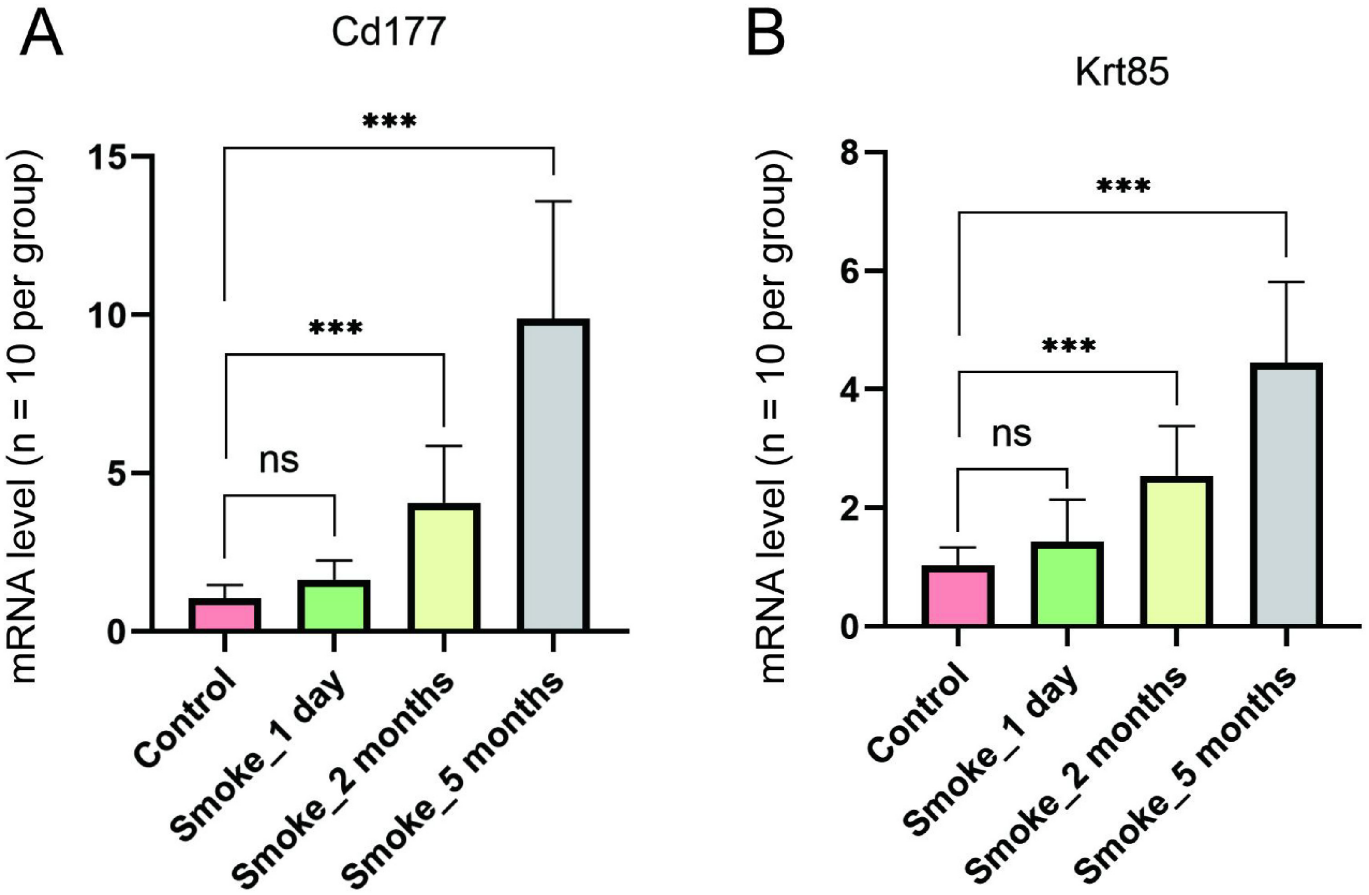
qPCR validation of mRNA expression levels of Cd177 and Krt85 in lung tissues across different durations of cigarette smoke exposure (*n* = 10 per group). **(A)** Relative mRNA expression levels of Cd177 in control, smoke exposure for 1 day, 2 months, and 5 months. **(B)** Relative mRNA expression levels of Krt85 in control, smoke exposure for 1 day, 2 months, and 5 months. Statistical significance was determined using one-way ANOVA followed by Tukey’s multiple-comparison test. ns, not significant (*p* ≥ 0.05); ***p < 0.001 (highly significant).

As shown in Figure 8, qPCR results further validated the expression patterns of the key candidate genes during cigarette smoke exposure. These analyses were performed using lung tissue samples from wild-type (WT) C57BL/6 mice (6–8 weeks old, male), with *n* = 10 biological replicates per group and at least three technical replicates per sample. Mice were exposed to a standardized medium-dose cigarette smoke protocol using a controlled exposure system, as described in detail in section 2.7. Compared with the control group, Cd177 expression was significantly increased in the 2- and 5-month smoke exposure groups (both *P* < 0.001), whereas only a non-significant upward trend was observed in the 1-day exposure group (*P* > 0.05), indicating that marked activation of Cd177 predominantly occurs during the intermediate-to-late stages of sustained smoke exposure (Figure 8A). Similarly, the mRNA expression level of Krt85 was significantly higher in the 2- and 5-month smoke exposure groups compared with the control group (both *P* < 0.001), while no significant difference was detected between the 1-day exposure group and controls (*P* > 0.05) (Figure 8B). These findings demonstrate that Krt85 upregulation is also time dependent, with progressively increased transcriptional levels accompanying prolonged cigarette smoke exposure.

### 3.8 Exploratory validation in a human COPD dataset

To further explore the translational relevance of the candidate genes, we analyzed the human COPD transcriptomic dataset GSE47460. In the revised analysis, samples from the GPL14550 platform were used, and interstitial lung disease samples were excluded. Because GSE47460 contains patients with heterogeneous COPD severity, we performed an exploratory severity-stratified analysis focusing on GOLD 4 very severe COPD samples compared with controls. In this severity-stratified analysis, CD177 and KRT85 showed increased expression in GOLD 4 COPD samples compared with controls after adjustment for age, sex, and smoking status. The expression patterns and exploratory discriminative analysis are shown in Supplementary Figure S3, and the adjusted differential expression results are provided in Supplementary Table S8. These findings suggest that the human expression signal of CD177 and KRT85 may be more evident in severe COPD phenotypes rather than uniformly present across all COPD samples. Therefore, the human dataset analysis should be interpreted as exploratory and severity-dependent, rather than as definitive validation of CD177 and KRT85 as general diagnostic biomarkers for COPD.

## 4 Discussion

In this study, we employed a time-series transcriptomic approach to comprehensively characterize lung gene expression alterations induced by cigarette smoke at different exposure stages and to elucidate their associations with COPD-related chronic airway inflammation. Our results demonstrate that short-term exposure (1 day) is sufficient to trigger acute transcriptional responses in lung tissue, primarily involving oxidative stress defense and early inflammatory activation. During the intermediate exposure stage (2 months), inflammatory responses become further amplified, accompanied by prominent changes in genes associated with tissue remodeling. In contrast, prolonged and sustained exposure (5 months) leads to consolidation of a chronic inflammatory state, with profound effects on immune dysregulation and epithelial remodeling. These findings are highly consistent with the pathophysiological progression of COPD, which is characterized by a gradual transition from early, potentially reversible inflammatory responses to persistent and largely irreversible chronic inflammatory injury. In the following sections, we discuss the major findings in detail by integrating specific differentially expressed genes and enriched pathways.

Our transcriptomic data indicate that pulmonary inflammatory pathways remain persistently activated with increasing duration of cigarette smoke exposure. Notably, the nuclear factor-κB (NF-κB) signaling pathway exhibited a consistent upregulation trend across all exposure time points, representing a canonical inflammatory pathway induced by cigarette smoking (13). Activation of NF-κB leads to enhanced transcription of multiple pro-inflammatory genes, including those encoding inflammatory mediators such as tumor necrosis factor-α (TNF-α), interleukin-1β (IL-1β), and interleukin-6 (IL-6), as well as neutrophil-attracting chemokines such as CXCL1/KC and CXCL8/IL-8 (14). The elevated expression of these mediators provides a plausible explanation for the marked infiltration of neutrophils and macrophages observed in the long-term smoke exposure groups. Neutrophils are key effector cells in COPD airway inflammation. Driven by chemotactic factors such as IL-8, neutrophils accumulate in the lung and release proteolytic enzymes, including elastase, myeloperoxidase, and neutrophil elastase, which contribute to damage of the airway and alveolar walls. Consistent with this pathological feature, our results showed significant upregulation of neutrophil-associated genes in the 5-month exposure group compared with controls, among which Cd177 was particularly notable. CD177 is a glycoprotein highly expressed on the surface of neutrophils and is closely involved in neutrophil adhesion and transendothelial migration (15). Previous studies have reported that in cigarette smoke-induced pulmonary inflammation models, CD177 facilitates neutrophil transmigration across the vascular endothelium, potentially through interactions with endothelial PECAM-1, thereby promoting neutrophil extravasation into lung tissue (16). Accordingly, the upregulation of Cd177 observed in our long-term exposure group suggests enhanced neutrophil recruitment under sustained cigarette smoke stimulation, with CD177 potentially acting as a key mediator of this process. Supporting this notion, a recent study reported increased numbers of circulating neutrophils with altered activation states in patients with early-stage COPD (17). Taken together, these findings suggest that elevated pulmonary CD177 expression may reflect neutrophil-associated inflammatory activity but may also amplify neutrophil-driven inflammatory circuits, thereby contributing to the maintenance and persistence of chronic inflammation in COPD. However, in human COPD datasets, CD177 exhibited heterogeneous performance, showing moderate discriminative ability in GSE47460 but limited performance in other cohorts. This suggests that while CD177 may reflect neutrophil-driven inflammation, its diagnostic value in human COPD remains context-dependent.

Cigarette smoke-induced oxidative stress spans multiple exposure stages and exerts profound effects on pulmonary barrier integrity as well as regenerative and reparative capacity. Our results indicate that even short-term smoke exposure is sufficient to trigger classical antioxidant defense responses, including upregulation of detoxifying and antioxidant enzyme genes downstream of the Nrf2 pathway, such as Ho-1 and Nqo1, reflecting an early activation of protective mechanisms against acute oxidative stress. However, with prolonged exposure, these antioxidant defenses may become progressively overwhelmed, leading to increasingly evident cumulative damage caused by excessive reactive oxygen species (ROS) (4).

Oxidative stress not only directly damages cellular lipids, proteins, and DNA, but also amplifies tissue injury through activation of inflammatory cascades. For example, ROS can activate transcription factors such as NF-κB and AP-1, thereby inducing the production of pro-inflammatory cytokines and chemokines and forming a positive feedback loop with inflammatory signaling pathways (18). More importantly, oxidative stress accelerates cellular senescence and aberrant cell death processes in pulmonary parenchymal cells. In the transcriptomic profiles of our long-term exposure group, altered expression of genes associated with apoptosis and regulated necrosised expression necroptosis—isropd regulated necrosised expression of genesposure reshapes cell death modalities in lung tissue.

Notably, the emerging concept of necroptosis has gained increasing attention in the context of COPD pathogenesis. Necroptotic death of epithelial cells leads to the release of intracellular damage-associated molecular patterns (DAMPs) and inflammatory mediators, thereby further amplifying inflammatory responses (19). A recent review by Hikichi et al. highlighted that cigarette smoke can induce defects in mitophagy, thereby triggering necroptosis and causing epithelial cell death accompanied by excessive inflammatory signal release, ultimately resulting in a sustained “inflammatory storm”-like tissue injury (5). Our data are consistent with this notion: even after prolonged exposure, inflammatory signals and tissue injury markers in the lung remained elevated, which may be attributable to necroptosis and the “inflammatory memory” associated with cellular senescence.

In addition, our results revealed downregulation of several genes involved in cell proliferation and regeneration in the long-term exposure group, such as proliferating cell nuclear antigen (PCNA). This finding suggests that chronic cigarette smoke exposure not only injures alveolar epithelial and endothelial cells, but also suppresses the proliferative and differentiation potential of resident stem and progenitor cell populations, thereby impairing normal tissue repair processes. In other words, under repeated smoke insults, lung tissue may enter a state in which damage exceeds repair capacity: persistent tissue destruction mediated by proteases and oxidative stress is not adequately counterbalanced by effective regeneration, ultimately leading to progressive alveolar destruction, airway wall remodeling, and fibrosisn other words, under repeated smoke insults, lung tissue may enas a critical driving force in this process and is therefore considered an important therapeutic target in COPD. Accordingly, our study underscores the intertwined roles of oxidative stress and chronic inflammation in smoke-induced lung injury and suggests that therapeutic strategies aimed at simultaneously restoring redox balance and suppressing chronic inflammation may represent effective approaches for intervening in cigarette smoke-related pulmonary damage.

Through integrative bioinformatic analyses and comparison with existing literature, we identified several differentially expressed genes that exhibited persistent and significant temporal changes, including Cd177 and Krt85. These genes may play important roles in COPD-related chronic inflammation and tissue remodeling. Below, we discuss the potential functions and mechanisms of these representative genes in the context of previous studies.

Cd177 is predominantly expressed on neutrophils. Functionally, CD177 serves as a key mediator of neutrophil adhesion and transendothelial migration by interacting with endothelial PECAM-1, thereby facilitating neutrophil extravasation into inflamed tissues (16). In our study, Cd177 expression was markedly elevated in the intermediate- and long-term smoke exposure groups, suggesting enhanced neutrophil infiltration and activation within the lung. Given that prominent neutrophil accumulation is a well-recognized pathological feature of the bronchial mucosa in patients with COPD, our findings support the notion that CD177 represents one of the critical “switches” driving neutrophil-mediated inflammation in COPD. Accordingly, CD177 may function as a nodal molecule linking inflammatory signaling with immune cell trafficking. Its role in COPD-associated inflammation therefore warrants further investigation. From a therapeutic perspective, blockade of CD177 or interference with CD177-mediated adhesion processes may have the potential to attenuate excessive neutrophil recruitment and thereby alleviate airway inflammation in COPD.

Krt85 (keratin 85), a member of the hair keratin family, is not normally expressed in healthy lung tissue. In contrast, we detected aberrant expression of Krt85 following chronic cigarette smoke exposure, suggesting a potential association with abnormal airway epithelial differentiation or squamous metaplasia. Although there are currently no direct reports describing the function of KRT85 in the lung, analogous alterations in keratin expression—such as KRT6 and KRT16/17, a member of the hair keraoking-related squamous epithelial lesions of the airway (20). Upregulation of Krt85 may therefore reflect an enhanced tendency of basal epithelial cells toward keratinizing differentiation, leading to alterations in epithelial barrier structure and compromised function. Such changes may render the airway epithelium more vulnerable to repeated inflammatory injury. In addition, atypical keratin expression or keratin fragments may act as danger-associated signals capable of activating innate immune surveillance mechanisms.

Thus, although the precise role of KRT85 in lung biology remains to be elucidated, its aberrant appearance itself may serve as a molecular indicator of cumulative smoke-induced injury. In our murine model, Krt85 expression demonstrated a relatively high area under the curve (AUC) for discriminating long-term smoke exposure from controls. However, in the exploratory human COPD analysis, KRT85 showed only limited discriminative performance, indicating restricted translational applicability. The exploratory GSE47460 analysis further suggests that the human expression signal of CD177 and KRT85 may be severity-dependent rather than uniformly present across all COPD samples; therefore, these genes should be regarded as murine model-derived candidate markers with limited and heterogeneous translational support in human COPD. Further functional studiesd KRT8as *in vitro* modulation of KRT85 expression to assess epithelial cell behavior support in human to clarify this issue.

Taken together, these key differentially expressed genes outline a coherent network of smoke-driven chronic inflammation. Cigarette smoke initially triggers acute inflammatory responses through oxidative stress and pro-inflammatory signaling; subsequently, immune cells—including neutrophils, macrophages, and lymphocytesignaling; subsequently, immune cells—including neutrophils, macrophages, atory mediators that cause tissue injury and epithelial barrier disruption. Damaged epithelial and structural cells then release danger signals and chemotactic factors (such as S100 proteins, IL-33, CXCL8, and CCL20), further amplifying immune cell recruitment and driving aberrant epithelial differentiation, including squamous metaplasia. Meanwhile, oxidative stress accelerates cellular senescence and dysregulated cell death, rendering inflammation refractory to resolution and allowing it to persist even after smoking cessation (5). These interwoven processes gradually transform acute, potentially reversible injury into chronic, irreversible damage, ultimately giving rise to the characteristic pathological features of COPD, including small airway inflammation and fibrosis, parenchymal destruction, and emphysema. By capturing these molecular signals in a time-resolved manner, our study provides a dynamic framework for understanding the progressive pathogenesis of COPD.

The findings of the present study are largely consistent with those of many previous investigations in terms of overall direction, while also revealing several distinctive features. First, we confirmed the recapitulation of multiple canonical COPD-related pathways in the animal model, including chronic inflammation, oxidative stress, and protease-antiprotease imbalance (21). Importantly, our time-series design uncovered dynamic changes in these pathways over the course of smoke exposure: some genes were rapidly upregulated at early stages and subsequently declined in an adaptive manner, whereas others became progressively more prominent with cumulative exposure.

This dynamic pattern is in line with observations reported by Miller et al. in their RNA sequencing study, in which 1 day of cigarette smoke exposure induced only modest transcriptional changes, whereas a substantial number of genes were significantly altered after 7 days, followed by additional pathway-specific reversals or compensatory regulation during prolonged exposure over several months (9). We observed a similar phenomenon in our data; for example, genes involved in oxidative stress defense were upregulated during the acute phase but declined during chronic exposure, whereas genes associated with mucus production showed the opposite trend. These findings suggest the presence of an “stress-decompensation” process in response to sustained cigarette smoke exposure. Initially, endogenous protective mechanisms are mobilized to counteract injury; however, with prolonged exposure, these defenses weaken or become exhausted, allowing injury-related processes to predominate. This temporal transition carries important biological implications, highlighting the critical role of intervention timing. Enhancing endogenous protective responses and mitigating initial inflammation at early stages may prevent the establishment of self-perpetuating inflammatory circuits. In contrast, once the system enters a decompensated state—such as in individuals with long-term, unabated smoking leading to established COPDted smoking leading the system enters a decompensated state—such protective reour findings reinforce the importance of early intervention and are concordant with epidemiological evidence demonstrating that early smoking cessation substantially reduces the risk of developing COPD. By capturing these temporal dynamics, our study provides mechanistic support for preventive strategies that prioritize early-stage modulation of smoke-induced lung injury.

The findings of this study provide several clinically relevant implications for the prevention and management of COPD. From a biomarker perspective, several genes identified in our analysis, such as CD177, KRT85, and CCL20, may have potential as candidate markers for COPD-related inflammatory processes rather than definitive diagnostic biomarkers. In particular, CD177 may serve as a potential indicator of neutrophil-driven airway inflammation. However, given the heterogeneous performance observed across human COPD datasets, the diagnostic value of CD177 requires further validation in larger and well-characterized clinical cohorts. If future studies confirm that CD177 levels are elevated in bronchoalveolar lavage fluid or sputum samples from smokers and correlate with the degree of neutrophilic inflammation, this marker could provide an objective measure of pulmonary inflammatory burden in smokers. These observations are further supported by previous transcriptomic studies in human COPD. For example, Liu et al. (22) performed RNA sequencing analysis in peripheral blood samples from COPD patients and identified widespread dysregulation of coding and noncoding RNAs, along with the construction of RNA regulatory networks. Notably, their gene set enrichment analysis revealed significant involvement of RNA metabolic and processing pathways, suggesting that COPD progression is associated with complex transcriptional and post-transcriptional regulatory alterations. Although the present study is based on a murine time-series smoke exposure model, the convergence of these findings indicates that inflammation-related genes identified here may represent conserved molecular features associated with COPD pathogenesis across species. This cross-validation with human transcriptomic data further strengthens the potential translational relevance of our candidate biomarkers (22).

Similarly, aberrant expression of KRT85, if detectable in airway biopsy specimens or exfoliated airway epithelial cells, may reflect abnormal epithelial differentiation or squamous metaplastic changes in the airway. Given the central role of epithelial remodeling in COPD pathogenesis, such markers could complement existing clinical and imaging assessments in identifying early structural alterations.

From a therapeutic standpoint, our findings support several existing or emerging treatment strategies for COPD. Notably, the IL-17 signaling pathway was repeatedly implicated in chronic inflammation across our models. Biological agents targeting this pathway, including anti-IL-17 monoclonal antibodies and IL-17 receptor A antagonists, have already demonstrated efficacy in asthma and psoriasis and may hold promise for subsets of COPD patients with pronounced inflammatory phenotypes. In addition, based on the central role of oxidative stress highlighted in our study, we emphasize the importance of enhancing Nrf2-mediated antioxidant defenses. Several Nrf2 activators, such as bardoxolone methyl and hematoxylin-related compounds, have shown potential in preclinical studies to attenuate cigarette smoke-induced lung injury (23).

Furthermore, our data suggest that preserving airway epithelial integrity may alleviate inflammation and slow COPD progression. Accordingly, therapeutic strategies aimed at protecting the mucosal barrier or promoting epithelial repair merit further investigation. These may include supplementation with epithelial growth factors (e.g., keratinocyte growth factor, KGF) or regenerative approaches such as stem cell-based therapies to repair epithelial damage.

Taken together, our study supports a “whole-course intervention” framework for COPD. During early exposure stages, therapeutic efforts should prioritize antioxidant and anti-inflammatory strategies to prevent the establishment of persistent inflammatory circuits. At intermediate and advanced stages, combined approaches targeting inflammatory mediators while simultaneously promoting tissue repair may be required to limit small airway remodeling and emphysema progression. This stage-specific, multi-target intervention concept aligns well with the heterogeneous and progressive nature of COPD and warrants further validation in future experimental studies and clinical trials.

Despite the meaningful findings obtained in this study, several limitations should be acknowledged. First, this work was conducted using a mouse model, and interspecies differences in lung anatomy, inflammatory responses, and susceptibility to cigarette smoke may limit the direct translation of these findings to human COPD. Second, microarray-based analyses have inherent limitations in sensitivity and specificity, which may result in the omission of genes with subtle but biologically relevant expression changes. In addition, transcriptional alterations do not necessarily reflect corresponding changes at the protein or functional level, and although qPCR validation was performed for selected genes, direct validation at the protein level was not included. Third, as this study was based on bulk lung tissue transcriptomic data, the observed gene expression patterns may be influenced by changes in cell-type composition, particularly immune cell infiltration during cigarette smoke exposure. To address this issue, we performed a marker-based proxy score analysis for major immune cell populations and incorporated these scores into sensitivity analyses. The results demonstrated that the main transcriptomic patterns and key candidate genes remained largely consistent after adjustment, suggesting that the identified molecular signatures are not solely driven by shifts in cellular composition. Nevertheless, we acknowledge that more refined approaches, such as single-cell RNA sequencing or computational deconvolution methods (e.g., CIBERSORTx), would provide a more precise characterization of cell-type-specific transcriptional regulation. Fourth, the integration of datasets from different microarray platforms (Affymetrix and Agilent) may introduce systematic bias due to differences in probe design, detection sensitivity, and dynamic range. Although our use of intersecting differentially expressed genes enhances robustness by focusing on consistently altered signals across datasets, the absence of dedicated cross-platform normalization (e.g., MatchMixer) remains a limitation. Future studies incorporating unified normalization frameworks and multi-omics validation, including proteomic and functional assays, will be important to further strengthen and extend these findings. Finally, the predictive model was developed and evaluated using the same dataset following feature selection, which may introduce selection bias and overfitting and thus potentially inflate the reported AUC values.

## 5 Conclusion

This study systematically characterized cigarette smoke-associated transcriptional responses across a murine exposure time series by integrating public datasets and experimental validation. We identified a stable set of inflammation-related candidate genes, with CD177 and KRT85 emerging as representative nodes linked to neutrophil-associated inflammation and epithelial remodeling in experimental models. These findings provide a transcriptomic framework for understanding smoke-induced chronic airway inflammatory responses and highlight candidate molecular modules for future mechanistic investigation. Further validation in larger, independent, and clinically well-characterized human cohorts is required before these genes can be considered clinically applicable biomarkers.

## Data Availability

The datasets presented in this study can be found in online repositories. The names of the repository/repositories and accession number(s) can be found at: https://www.ncbi.nlm.nih.gov/, GSE18344; https://www.ncbi.nlm.nih.gov/, GSE132661; and https://www.ncbi.nlm.nih.gov/, GSE47460.

## Footnotes

1
https://www.ncbi.nlm.nih.gov/geo/
