# Supplementary figures and images for: Time-series transcriptomic analysis of cigarette smoke–associated lung responses reveals COPD-related inflammatory and epithelial remodeling modules in murine models
Source: Front Med (Lausanne). 2026 Jun 24;13:1785075. doi: 10.3389/fmed.2026.1785075 (PMC13341621; doi:10.3389/fmed.2026.1785075)

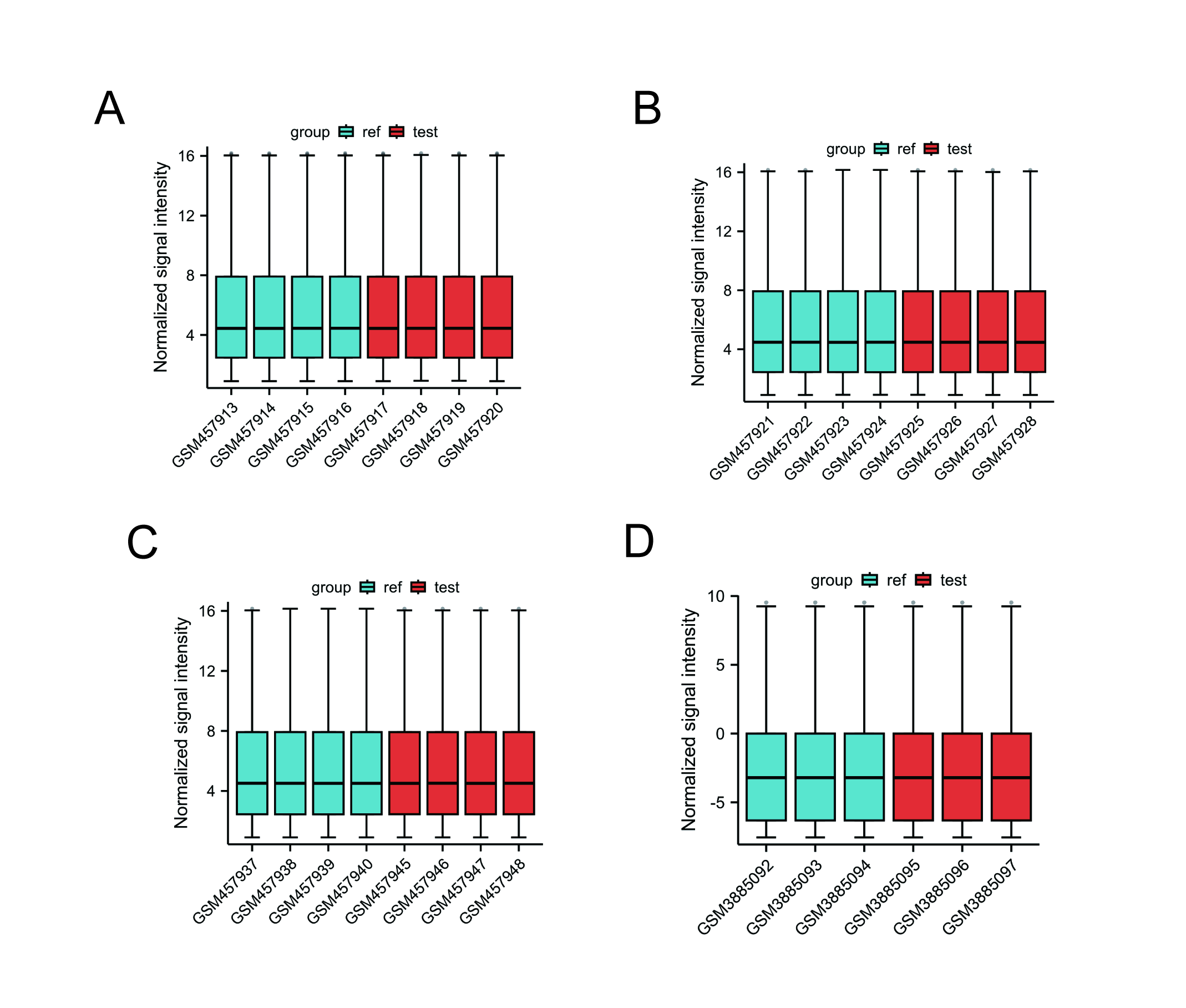

Supplement: Supplementary file 1 [file Image_1.tif]

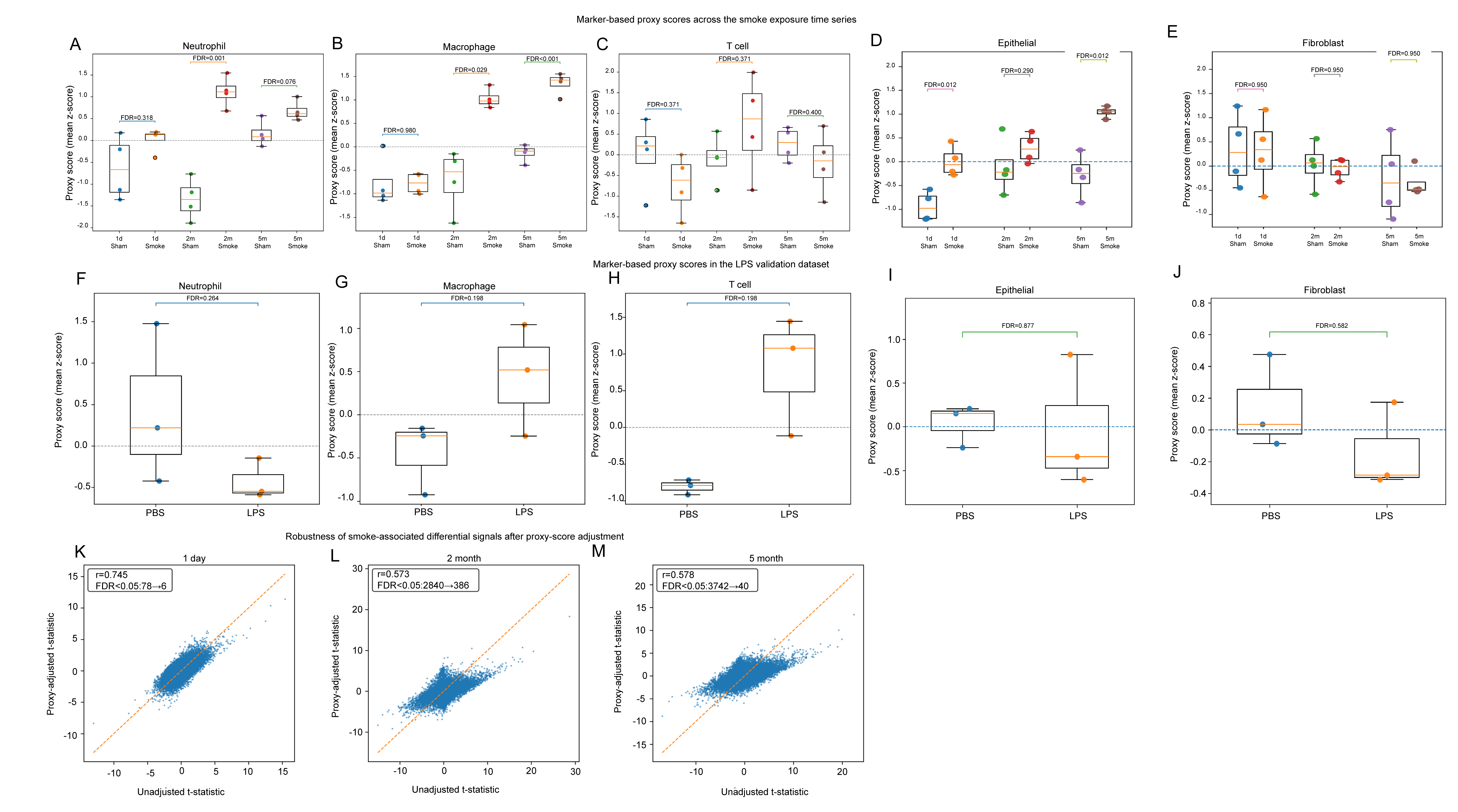

Supplement: Supplementary file 2 [file Image_2.tif]

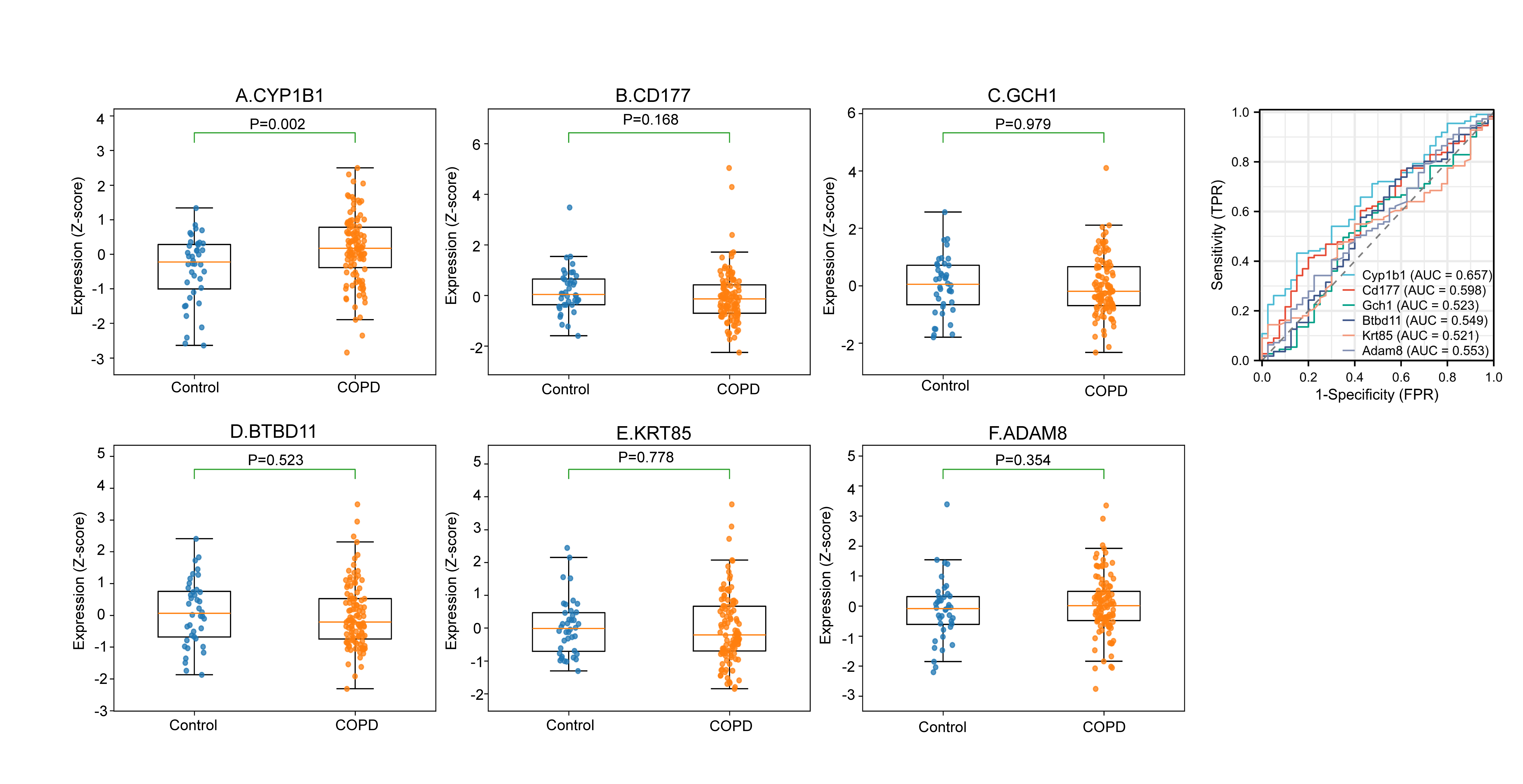

Supplement: Supplementary file 3 [file Image_3.tif]
