# Supplementary material for: Time-series transcriptomic analysis of cigarette smoke–associated lung responses reveals COPD-related inflammatory and epithelial remodeling modules in murine models
Source: Front Med (Lausanne). 2026 Jun 24;13:1785075. doi: 10.3389/fmed.2026.1785075 (PMC13341621; doi:10.3389/fmed.2026.1785075)
Supplement: Supplementary file 4 [file Supplementary_file_1.pdf]

Supplementary Figure S1. Boxplots of normalized signal intensity across samples at each time point, demonstrating consistent data distribution after normalization. (A) 1-day exposure group. (B) 2-month exposure group. (C) 5-month exposure group. (D) LPS-induced chronic airway inflammation model.

Supplementary Figure S2. Marker-based cellular proxy score analysis and robustness assessment after proxy-score adjustment.

Note: (A-E) Marker-based proxy scores for neutrophils, macrophages, T cells, epithelial cells, and fibroblast/stromal cells across the cigarette smoke exposure time series. Proxy scores were calculated as the mean Z-score of representative marker genes for each cell type. Epithelial proxy scores exhibited stage-dependent variation in the smoke exposure model, with significant differences observed at 1 day and 5 months, whereas fibroblast/stromal proxy scores did not show significant changes across exposure stages. (F-J) Marker-based proxy scores for neutrophils, macrophages, T cells, epithelial cells, and fibroblast/stromal cells in the LPS-induced chronic airway inflammation validation dataset. Epithelial and fibroblast/stromal proxy scores did not differ significantly between PBS and LPS groups. (K-M) Comparison between unadjusted and proxy-score-adjusted differential expression statistics at 1 day, 2 months, and 5 months of cigarette smoke exposure. The proxy-score-adjusted models incorporated immune-cell proxy scores and non-immune proxy scores, including epithelial and fibroblast/stromal scores. Although the overall differential expression patterns remained broadly comparable after adjustment, the stage-dependent epithelial proxy-score variation suggests that epithelial cell composition or epithelial cell-state changes may partially contribute to the differential gene expression signals detected in bulk lung tissue.

Supplementary Figure S3. Exploratory severity-stratified validation of candidate genes in the GSE47460 human COPD transcriptomic dataset.

Expression data were derived from the GPL14550 platform. Interstitial lung disease samples were excluded, and GOLD4 very severe COPD samples were compared with control samples. Z-score transformation was used only for visualization and was not used for statistical testing. Differential expression of CD177 and KRT85 was evaluated using linear models adjusted for age, sex, and smoking status.

(A – F) Expression patterns of the six candidate genes in control and GOLD4 COPD samples. (G) Exploratory ROC analysis of the six candidate genes in the same severity-stratified subset. The ROC analysis was used only to describe exploratory discriminative performance and was not used as definitive evidence of clinical diagnostic validity.
